# A multi-omics approach exploring the gut-liver axis following combined radiation exposure and burn injury in a Sinclair minipig model

**DOI:** 10.1038/s41598-025-24946-0

**Published:** 2025-11-20

**Authors:** Timothy S. Horseman, Babita Parajuli, Veda Murthy, Gregory P. Holmes-Hampton, Gauthaman Sukumar, Clifton L. Dalgard, Joseph A. Anderson, David M. Burmeister

**Affiliations:** 1 School of Medicine, Uniformed Services University of the Health Sciences, Bethesda, MD 20814 USA; 2 Armed Forces Radiobiology Research Institute, Uniformed Services University of the Health Sciences, Bethesda, MD 20814 USA; 3 The American Genome Center, Center for Military Precision Health, Uniformed Services University of the Health Sciences, Bethesda, MD 20814 USA; 4 Comparative Pathology Division, Department of Laboratory Animal Resources, Uniformed Services University of the Health Sciences, Bethesda, MD 20814 USA

**Keywords:** Microbiology, Pathogenesis

## Abstract

While radiation and burn injury have distinct local and systemic effects, they both negatively impact intestinal permeability/function. In a nuclear attack, combined thermal burns and radiation exposure (e.g., combined injury (CI)) would be a dominant injury pattern. Despite this, how burns affect gastrointestinal acute radiation syndrome, and vice versa, has been largely unstudied. Next-generation sequencing has revealed a strong bidirectional link between the liver and gut. Here, we used a porcine model to evaluate the impact of burn, radiation, and CI on the gut microbiota and liver transcriptomics to determine if this link exists in these injury patterns. Sinclair minipigs were randomly divided into three groups: burn (*n* = 8), hemibody radiation (*n* = 7), and CI (*n* = 8). Animals were monitored for 14 days with longitudinal rectal swab and blood collection. CI increased weight loss, diarrhea, inappetence and lethargy compared to either injury pattern alone. Jejunum histology revealed increased mucosal apoptosis in burn and CI groups compared to radiation. CI led to elevated levels of NLRP3 and IL1β in the jejunum. Intestinal barrier disruption was indicated by decreasing circulating l-citrulline in CI which correlated inversely with intestinal-Fatty Acid Binding Protein. Bacteremia was elevated post-burn on day 1 and again from day 7–14 in burn and CI groups. Microbiome analysis showed phylogenetic shifts and differential abundance across groups with CI animals exhibiting distinct microbial signatures linked to intestinal dysfunction and liver injury. Liver RNA-seq revealed group-specific gene expression changes, with CI-altered pathways implicating immune responses and lipid metabolism, which were correlated with gut microbiota such as *Bacteroidaceae* and *Lachnospiraceae*. Taken together, we present a first-of-its kind large animal model of radiation combined injury to highlight the interplay between gut-liver axis disruptions and systemic injury responses.

**Supplementary Information:**

The online version contains supplementary material available at 10.1038/s41598-025-24946-0.

## Introduction

In a nuclear attack, combined injury (CI) such as radiation exposure and burns would become a dominant injury pattern. Although an estimated 60% of Hiroshima and Nagasaki atomic bomb casualties sustained radiation exposure accompanied by burn injury, the mechanistic basis of how burns affect acute radiation syndrome (ARS) and vice versa is largely unstudied^1^. CI has primarily been evaluated in mice with a variety of injury types (wounding, burns, hemorrhage, or infection) following radiation exposure. Evidence suggests CI is characterized by immune suppression, increased apoptosis, and multi-organ dysfunction (MOD) leading to higher mortality compared to a single insult or radiation exposure^1,2^.

Both severe burn injury (i.e., > 20% total body surface area (TBSA)) and ARS result in systemic reactions involving inflammation, oxidative stress, and cell death. In addition, each trauma type invokes unique physiological responses such as burn-induced hypermetabolism^3^, and radiation-induced DNA damage^4^. Furthermore, burn and irradiation both independently compromise gut barrier integrity, increasing bacterial translocation^5^. Subsequently, the risk of sepsis becomes more likely which is a major cause of morbidity and mortality following burn and gastrointestinal acute radiation syndrome (GI-ARS)^4,6^.

Beyond the critical role of the intestinal epithelium in gut health and bacterial translocation, prior studies highlight the gut’s systemic influence, describing it as a “motor” for critical illness with complex crosstalk to distant organs^7^. An important component to barrier integrity and host homeostasis are the commensal gut microbiota^8^. Alterations in the gut microbiota have been associated with intestinal dysbiosis and poor clinical outcomes following a variety of traumatic injuries and diseases^9–11^. Similar to the gut, the liver is vital for immune, inflammatory, and metabolic processes. Additionally, the gut and liver communicate bidirectionally with 80% of hepatic blood supply through the portal vein via the gut^12^. In chronic diseases, such as inflammatory bowel disease, a link between the gut microbiota and liver has been demonstrated^13^. However, limited data is available to elucidate the relationship following traumatic injury and how alterations could ultimately lead to systemic consequences complicating recovery^6^. Furthermore, the impact of CI on the gut microbiota remains to be addressed.

In the present study, our aim was to determine the effect of large total body surface area contact burns on the onset and progression of GI-ARS in the Sinclair minipig. We examined the clinical outcomes and molecular signatures of intestinal dysfunction with a primary focus on the gut microbiota-liver axis. This model represents the first large animal study which integrates a top down sequencing approach to examine the interaction of the gut microbiota and liver. Unlike previous rodent models that have focused on short-term survival, hematologic changes, or epithelial damage within 72 hours post-injury, our model evaluated persistent GI dysfunction and multi-organ interaction beyond the acute phase.

Importantly, swine allow for gut-liver systems-level analysis in a species with closer physiological similarity and bacterial taxonomy to humans. This approach allows for direct investigation of how CI alters host-microbe interactions, which remains poorly characterized even in models of burn or radiation injury alone.

We hypothesized that CI would increase intestinal dysfunction compared to either injury alone and be associated with a disrupted gut microbiome composition with reduced beneficial flora and increased opportunistic pathogens. Further, we hypothesized that the extent of this dysbiosis would affect ensuing hepatic function and transcriptomic alterations. These findings expand our knowledge of combined burn and radiation injury and provide new avenues for therapeutic targets in burns, GI-ARS, and CI.

## Materials and methods

### Ethics statement

An Association for Assessment and Accreditation of Laboratory Animal Care—International (AAALACI) accredited facility accredited was used to perform the study. The study was approved by the Institutional Animal Care and Use Committee (IACUC) of the Armed Forces Radiobiology Research Institute with all procedures in accordance with standard protocols and regulations. Additionally, our study complied with Animal Research: Reporting In Vivo Experiments (ARRIVE) guidelines.

### Animals and injury

Twenty-three male Sinclair minipigs (22.3 ± 5.1 kg) were obtained from Sinclair Bio Resources (Auxvasse, MO) and housed at least 72 h prior to any procedures. Sinclair minipigs were chosen in order to allow for both the utilization of a sexually mature model, as well as for accommodation with the size of the radiation field. Minipigs were randomly assigned to one of three injury patterns: thermal burns (*n* = 8), radiation exposure at 8 Gy (*n* = 7), or combined radiation and burns (CI, *n* = 8). All animals were anesthetized with an intramuscular (IM) injection of tiletamine-zolazepam (Telazol, 4–8 mg/kg), hair removed from the dorsum, flanks, and legs with a razor then an indwelling jugular catheter was placed prior to any injury as described previously^11^. Full thickness thermal burn wounds were created with brass blocks (e.g., 5 × 5 cm and 9 × 15 cm) maintained at 100 ± 0.2 °C applied to the skin for 30 seconds, this was repeated until approximately a 25% total body surface area burn was achieved. Burns were covered with silver sulfadiazine cream (Ascend Laboratories, Parsippany, NJ) and Ioban Antimicrobial dressings (3 M, St. Paul, MN). Hemibody irradiation of 8 Gy was performed as described previously, targeting distal to the diaphragm with a clinical linear accelerator (Elekta Infinity, Armed Forces Radiobiology Research Institute LINAC) delivering 4 MV photons with an approximate dose rate of 1.9 Gy/min^11^. In CI, irradiation was performed post-burn and animals were transported back to their home cages for recovery.

### Animal care and sample collection

Scoring of behavior and gastrointestinal symptoms was performed with body temperature and weight recorded daily as described previously^11^. Blood and rectal swabs were collected prior to injury (day 0) and on days 1, 2, 3, 7, 10, and 14 post-injury. Rectal swabs were stored at −80 °C until DNA isolation. Vacutainer tubes with Lithium Heparin (Becton Dickinson (BD), Franklin Lakes, NJ) and vacutainer tubes containing K2 EDTA (BD) were centrifuged at 20,000g for 15 minutes, plasma and sera were stored at −80 °C. After 14 days, animals were euthanized with sodium pentobarbital through the jugular catheter (Euthasol, 100 mg/kg, Virbac, Carros, France). Post-mortem tissues to include jejunum, liver, skin, and kidney were harvested then either snap-frozen, placed in RNALater (Invitrogen, Waltham, MA) and stored at −80 °C, or preserved in 10% formalin. Liver tissue was immediately homogenized in phosphate-buffered saline (PBS) for aerobic bacterial culture.

### Blood analyses

Clinical chemistry analyses were performed on the VITROS 350 (Ortho-Clinical Diagnostics, Raritan, NJ) evaluating markers of liver (alanine aminotransferase (ALT), aspartate aminotransferase (AST)), and kidney (blood urea nitrogen (BUN), and creatinine) function. Complete blood cell analyses including white blood cells, neutrophil, and lymphocytes were conducted using the Element HT5 Veterinary Hematology Analyzer (Heska, Loveland, CO). Bacteremia was assessed by plating approximately 0.1 mL of blood onto tryptic soy agar with 5% sheep blood (BA) and MacConkey agar (MAC) (Becton Dickinson) with plates incubated at 37°C overnight for colony forming unit (CFU) measurement. L-citrulline levels in sera were measured using High-Performance Liquid Chromatography on an Agilent 1200 system using an iHILIC Fusion column described previously^11^. ELISA kits were used to detect circulating levels of intestinal fatty acid binding protein (iFABP, MBS2501296, MyBioSource, San Diego, CA), C-Reactive Protein (CRP, NBP2-67253, Novus Biologicals, Centennial CO), and Syndecan-1 (LSBio, LS-F55926-1, Shirley, MA).

### Histology

Fixed samples were embedded in paraffin and cut into 5-µm sections. Slides were stained with standard hematoxylin and eosin (H&E) and a modified Gram stain procedure^14^. A blinded board-certified veterinary pathologist semi-quantitatively scored jejunum, liver, and kidney H&E slides with the following scale: 0 = normal; 1 = minimal; 2 = mild; 3 = moderate; 4 = marked; 5 = severe. Kidney sections were evaluated for tubular injury, glomerular changes, interstitial inflammation/fibrosis, edema, hemorrhage, casts, mineralization, and cellular infiltrates. Liver scoring included hepatocellular degeneration, necrosis, vacuolation, bile duct and hepatocyte hyperplasia, fibrosis, inflammation, congestion, hemorrhage, fatty change, amyloid, and thrombi. Jejunum was assessed for villar blunting, crypt loss, epithelial apoptosis, mucosal erosion, congestion, and inflammatory infiltrates. Immunohistochemistry (IHC) for Ki67 (1:200, MyBioSource), and TUNEL staining was performed (Abcam). Images were acquired with an Axio Scan.Z1 slide scanner (Zeiss Microscopy, White Plains NY), and high magnification images with a Lecia microscope. ImageJ (National Institutes of Health, Bethesda, MD) was used for semi-quantification of IHC and TUNEL staining, and measurement of villi length and re-epithelialization of skin sections.

### Protein analysis

For detection of biomarkers in tissue using ELISAs and western blotting, lysates were created by suspending cut tissues in diluent placed in Next Advance Eppendorf Lysis tubes (Next Advance, Troy, NY) with homogenization using the Bullet Blender 5E Gold (Next Advance). Western blot analysis was performed on jejunum and liver lysates for total proliferating cell nuclear antigen (PCNA; MyBioSource), Caspase-3 (Novus), Occludin (Sigma), Claudin-1 (Novus), E-Cadherin (Novus), NOD-, LRR- and pyrin domain-containing protein 3 (NLRP3, Sigma), Caspase-1 (LSBio), and interleukin-1 beta (IL-1β, Invitrogen) (Table S1). Protein (50–100 µg) was separated on 4–12% gels (Bio-Rad, Hercules, CA) with a semi-dry transfer to PVDF membranes (Bio-Rad). Secondary antibodies, IRDye 800CW or IRDye 680RD, were applied for visualization on the Odyssey infrared imaging system (LI-COR, Lincoln, NE). Beta-actin was used for normalization.

### Sequencing and bioinformatics for gut Microbiome and liver transcriptome

Rectal swab DNA was isolated using the QIAmp PowerFecal Pro kit (Qiagen, Germantown, MD). DNA was quantified using Qubit 4.0 (ThermoFisher). Library preparation was performed with 16S V4 region 515F–806R primers as described^11,15^. 16S V4 sequencing was performed on a NextSeq 500 using a Mid Output v2.5 2×150 kit (Illumina San Diego, CA). Bioinformatic analysis was performed as described previously^11^. Negative controls, including an extraction blank and a negative library control were included during processing to monitor for contamination. Briefly, nf-core/ampliseq (github.com/nf-core/ampliseq v2.7.0) was used for initial FASTQ quality processing of forward reads with DADA2 for feature table generation. The resulting feature table was imported into Quantitative Insights Into Microbial Ecology (QIIME2, version amplicon-2024.5), with taxonomy produced from a pretrained V4 classifier against Greengenes2 database. QIIME2 plugin fragment insertion SEPP was used for tree generation and subsequent diversity metric generation. ANCOM-BC, q2-composition, was performed for differential abundance of taxonomy and functional data. Taxa differential abundance threshold was set at *p* < 0.05 and LFC > 1.5.

After liver homogenization using the Bullet Blender (NextAdvance), RNA was isolated with the Direct-zol RNA Miniprep kit with TRI Reagent (Zymo Research, Orange, CA), and quantified using Qubit 4.0 (ThermoFisher). Briefly, 80 ng of total RNA was input using the RNA Prep with Enrichment + Exome panel (Illumina). Sequencing was performed on the NovaSeq X with the 1.5B 2×100 kit (Illumina). FASTQ files were processed using nf-core/rnaseq (v3.12.0; https://github.com/nf-core/rnaseq) with reads aligned to the pig genome, *susScr3*^16^. Gene counts from Salmon were used for downstream analyses in iDEP2.0^17,18^. False Discovery Rate (FDR) cutoff using Holm procedure for multiple testing correction was set for 0.05. Initial DNA and RNA extraction quantification was performed on Qubit 4.0 (ThermoFisher). Quality and quantity of libraries was determined by KAPA library quantification (Roche, Indianapolis, IN), and fragment analysis (Bioanalyzer 2100, Agilent, Savage, MD). Liver tissue from sham animals described previously were used as a reference control^11^. Sequences were deposited in the NCBI Sequence Read Archive.

### Statistics

Statistical evaluations were performed using Prism 10.3.1 (Graphpad Software, San Diego, CA) for clinical endpoints (e.g. weight loss, diarrhea, behavior, appetite), western blotting, ELISA, and alpha diversity data. Permutational analysis of variance (PERMANOVA) testing from QIIME2 was used for beta diversity metrics with Mantel testing for correlation to metadata variables. P values set as less than 0.05 were considered significant. All data is based on an *n* = 8 in burn and CI groups, and *n* = 7 for radiation. Results are expressed as mean ± SEM except for clinical outcomes and pathologist scoring which are represented as median and interquartile ranges. For the clinical endpoints, area under the curve analysis was performed. A two-way analysis of variance (ANOVA) with Tukey’s or Dunnett’s post-testing was used to determine the significance of injury and time. A one-way ANOVA or Kruskal-Wallis test was used for terminal study endpoints. Pearson correlation was used for longitudinal and terminal markers of intestinal dysfunction, inflammation, and injury response. Microbiome and liver associations were performed in R with metadata variables centered and scaled for Spearman’s correlation. Correlations between liver gene expression (R log transformed) and bacterial relative abundance were tested with Spearman’s correlation and Benjamini and Hochberg screened.

## Results

### Effect of combined burn and radiation injury on clinical outcomes

**Fig. 1:**
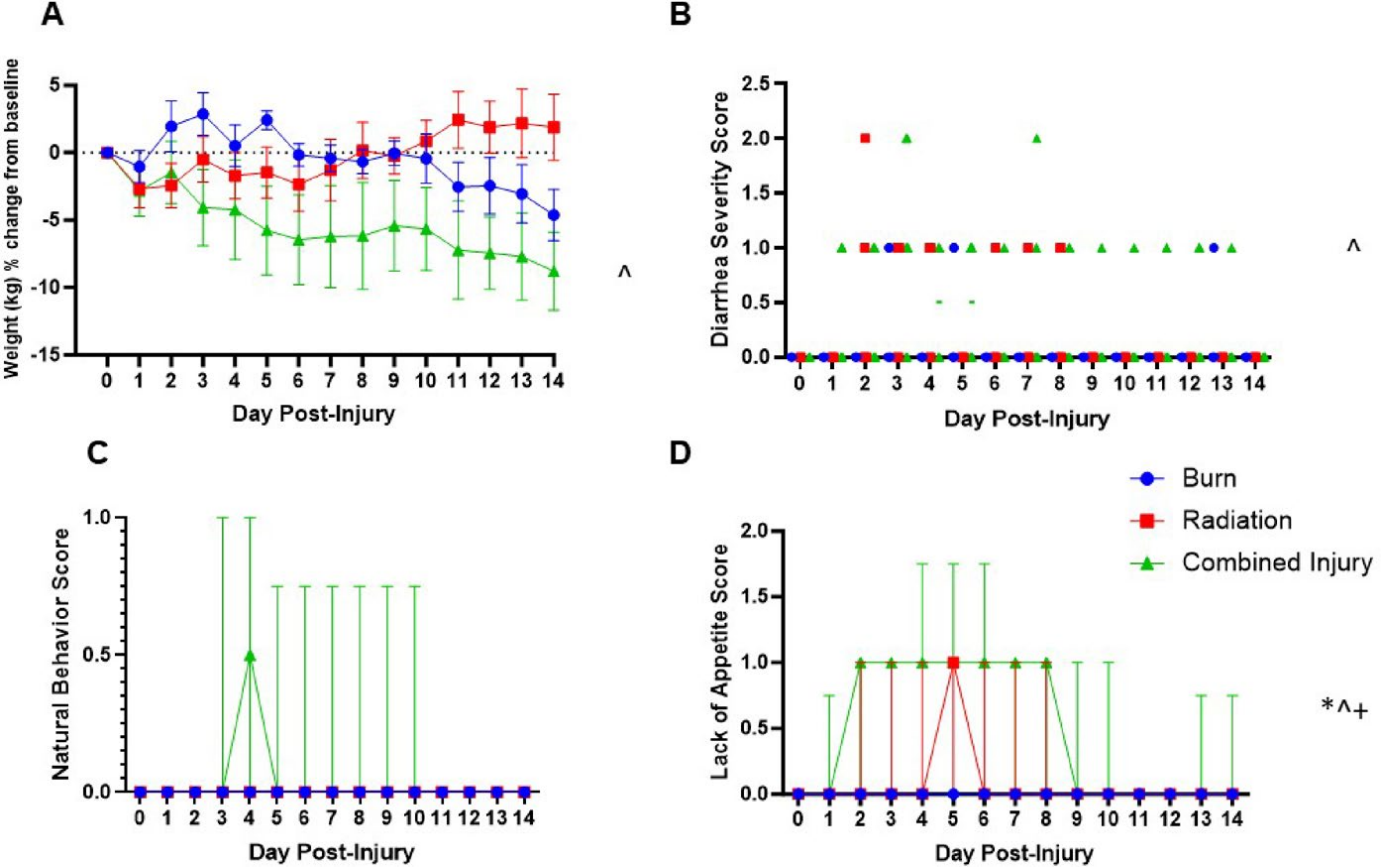
Effect of burn, radiation, and combined injury (CI) on clinical outcomes. Longitudinal data are shown as (**A**) percent weight change from baseline, (**B**) diarrhea prevalence and severity score, (**C**) natural behavior score, and (**D**) loss of appetite score. Area under the curve analysis revealed weight loss (*p* = 0.047) and diarrhea severity (*p* = 0.016) differed significantly between the CI and burn groups. There were also significant differences in appetite between groups (Burn v Rad, *p* = 0.016; Burn v CI, *p* < 0.0001; Radiation v CI, *p* = 0.002). No significant differences existed in minipig behavior among groups. Statistical significance between groups is represented as follows: * burn and radiation, ^ burn and CI, and + radiation and combined injury (*P* < 0.05). Burn (*n* = 8), radiation (*n* = 7), and CI (*n* = 8) at each timepoint. The color of each symbol represents the type of injury.

Thermal burns were not completely epithelized by day 14 with similar outcomes between CI and burn (Fig. S1). All animals survived the 14-day study. CI led to greater weight loss compared to burn (*p* = 0.047) or radiation (*p* = 0.062) alone. Additionally, CI resulted in significantly higher diarrhea prevalence than burns (*p* = 0.016), decreased appetite (Burn v CI, *p* < 0.0001; Radiation v CI, *p* = 0.002) with increased lethargy and aggression (Burn, *p* = 0.017; Radiation, *p* = 0.066) (Fig. 1A-D). Differential blood cell counts showed significant leukocytosis in the burn group compared to the radiation group post-injury (*p* < 0.0001–0.013) which is explained by increases in both neutrophils and lymphocytes (Fig. S2A-C). In CI, burn-induced changes in WBC counts initially appear suppressed by the effects of radiation until day 7 when WBC levels begin to rise until day 14 (Radiation v CI, *p* < 0.0001–0.001; Burn v CI, *p* = 0.004–0.043). All groups displayed transient acute kidney injury, with increases in blood urea nitrogen as well as creatinine, which was significantly greater in the CI group at day 2 (Fig S3A-B). Although renal dysfunction resolved by day 14, there were significant findings of increased renal tubular degeneration and necrosis in the radiation and CI groups compared to burn (Fig. S3C).

### Injury-induced GI epithelial damage

**Fig. 2:**
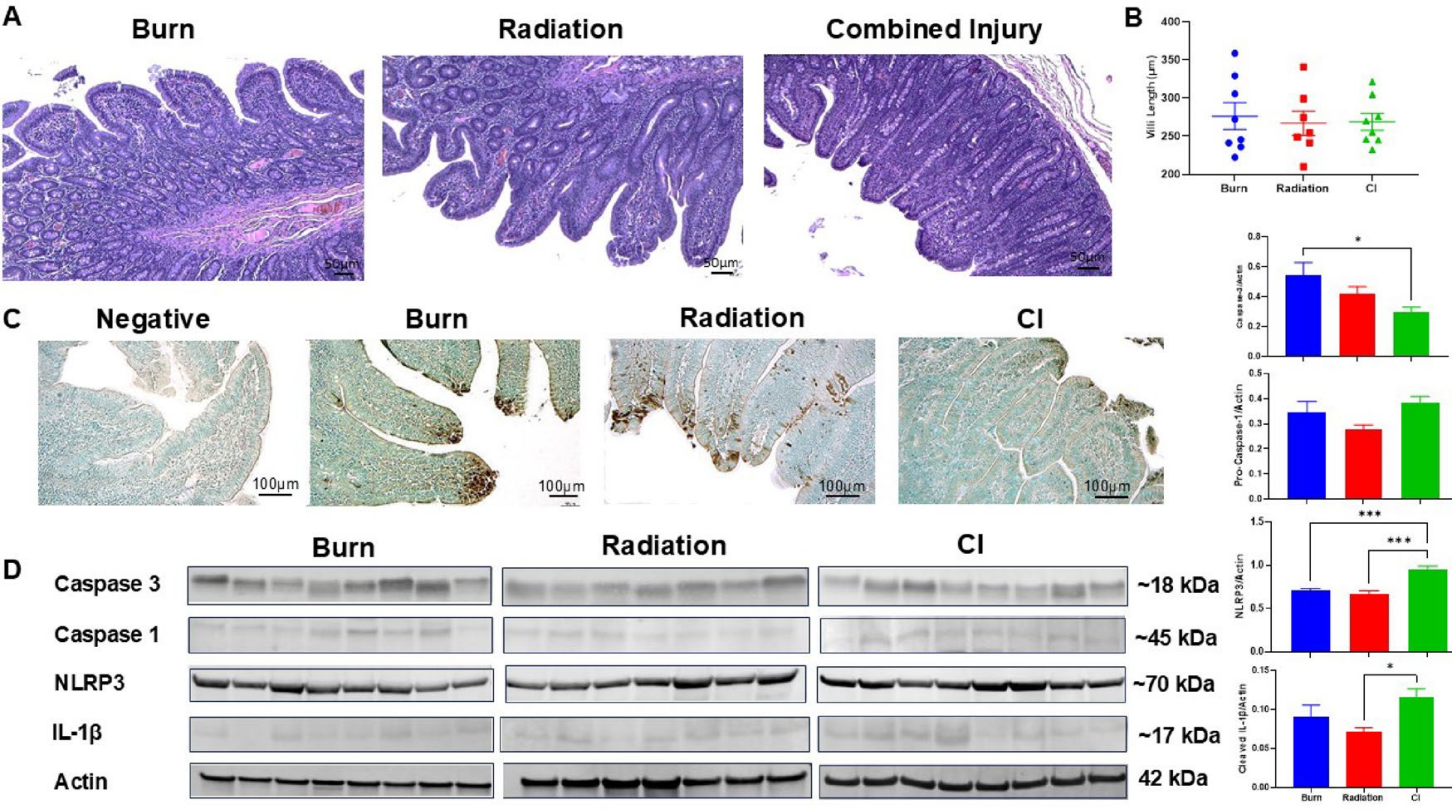
Histological analysis of injury-induced alterations to the jejunum. (**A**) Representative H&E stained images of jejunum from each injury pattern (scale bar 50 μm). (**B**) Measured villi length in µm, represent data points with mean ± SEM. (**C**) Immunohistochemistry of TUNEL staining with brown representing DNA fragmentation and green for normal cells. Representative 10x images taken with Leica microscope, scale at 100 μm. (**D**) Western blotting of Caspase-3 and components in the NLRP3 inflammasome activation pathway including NLRP3, pro-caspase-1 (Caspase-1), and cleaved interleukin-1 beta (IL-1β). Representative cropped Western blot images from independent replicate experiments are shown. Data are represented as mean ± SEM. Quantification of western blots are shown below the images. Burn (*n* = 8), radiation (*n* = 7), and CI (*n* = 8). *P < 0.05 and *** *P* < 0.001 (One-way ANOVA, Tukey’s post-testing).

Villi blunting across all groups was seen compared to our previously published sham villi lengths (458.46 ± 52.09 μm) for Sinclair minipigs (Fig. 2A-B)^16^. Pathologist scoring showed decreased mucosal cell apoptosis following CI (Fig. S4A), which was confirmed with TUNEL staining (Fig. 2C). Alternatively, no significant differences were observed in Ki67 IHC or PCNA western blotting amongst groups (Fig. S4B-C). However, caspase-3 post-burn was significantly elevated compared to CI (*p* = 0.028) (Fig. 2C-D). Pyroptosis and NLRP3 inflammasome activation, was then shown to be increased in CI with increased pro-caspase-1 and significantly higher NLRP3 protein levels (*p* = 0.004 vs. burn, *p* = 0.002 vs. radiation) as well as cleaved IL-1β (*p* = 0.043 vs. radiation, Fig. 2D).

**Fig. 3:**
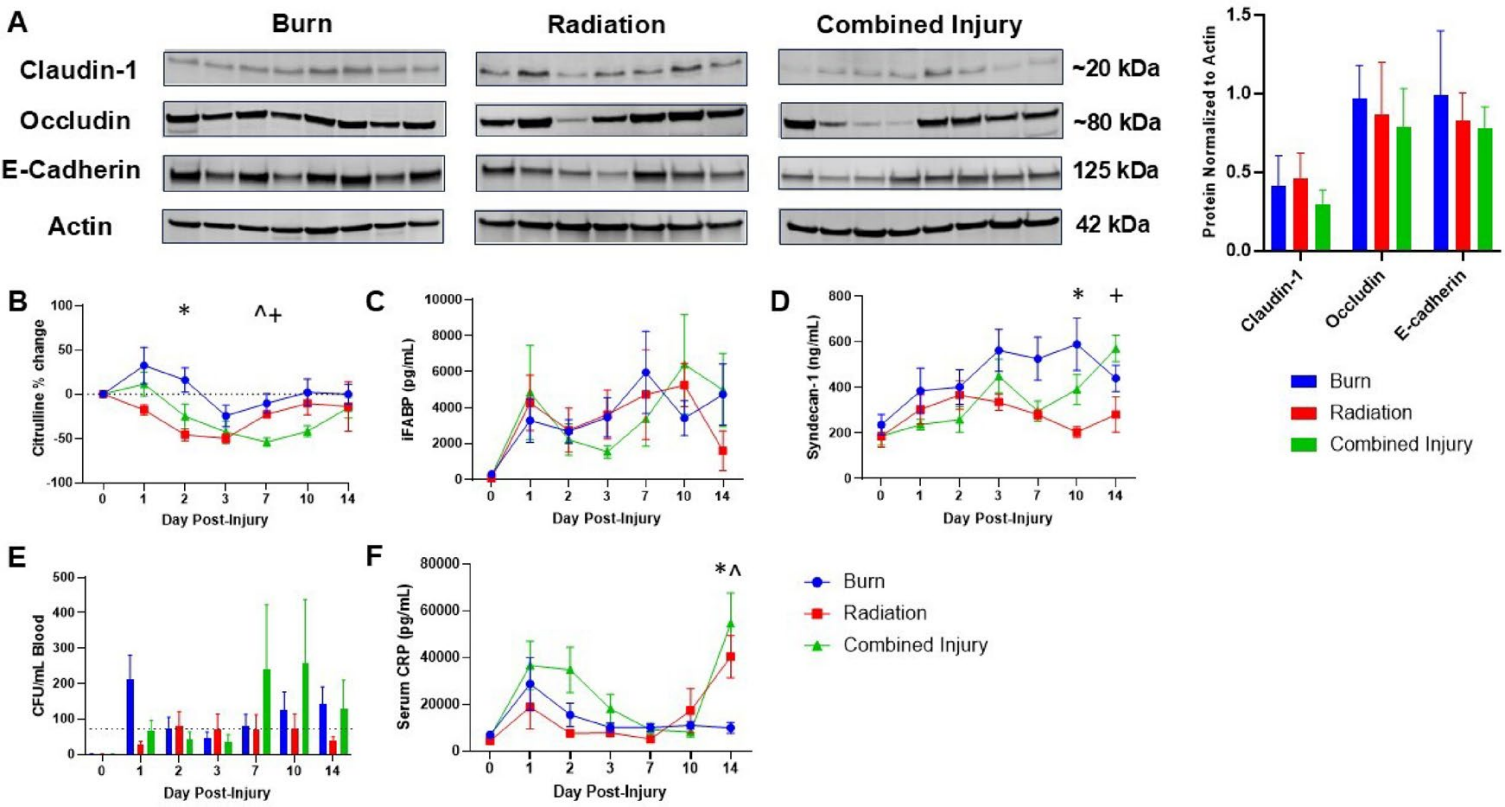
Gut epithelium integrity evaluated by Western blotting, detection of circulating biomarkers, and bacterial translocation. (**A**) Western blots showing jejunum tight junction proteins (claudin-1 and occludin) and an adherens junction protein (E-cadherin). Representative cropped Western blot images from independent replicate experiments are shown (One-way ANOVA, Tukey’s post-testing). (**B-F**) Circulating biomarkers related to intestinal dysfunction including l-citrulline shown as percent change from baseline, intestinal fatty acid binding protein (iFABP), syndecan-1, bacterial colony forming units per milliliter of blood (CFU/mL) and C-reactive protein (CRP) (Two-way ANOVA, Tukey’s post-testing). Statistical significance between groups is represented as follows: * burn and radiation, ^ burn and CI, and + radiation and combined injury (*P* < 0.05). Burn (*n* = 8), radiation (*n* = 7), and CI (*n* = 8) for each timepoint.

We next aimed to investigate epithelial barrier disruption by examining jejunal junction proteins and circulating biomarkers of intestinal dysfunction. Two-way ANOVA revealed a significant overall effect of injury on the expression of occludin, claudin-1, and E-cadherin (*p* < 0.0001), wherein CI animals consistently had lowest expression of these junctional markers. However, post-hoc testing revealed no statistically significant differences for any one target (Fig. 3A). L-citrulline (CIT) decreased regardless of the injury group (Fig. 3B). Irradiated animals had significantly decreased CIT than those burned on day 2 (*p* = 0.0064) with both reaching a low at day 3, while CI led to significantly lower levels (Burn, *p* = 0.012; Radiation, *p* = 0.005) which continued until day 14. I-FABP concentrations drastically increased post-injury, with no differences amongst the groups (Fig. 3C). Syndecan-1 levels also increased post-injury, with significantly higher levels in the burn group at day 10 (*p* = 0.029), and the CI group on day 14 (*p* = 0.029) (Fig. 3D).

This impaired barrier dysfunction was also associated with inflammatory changes, including bacteremia, especially in the CI group in week 2; however, there were no significant differences (Fig. 3E). Serum CRP also spiked following injury, and the chronic presence of radiation caused a second increase in CRP levels at day 14 in the radiation (*p* = 0.033), and CI (*p* = 0.026) groups compared to burn (Fig. 3F). Intestinal occludin levels showed a significant correlation with circulating CRP at day 14 (*p* = 0.035, *r* = 0.45) as well as bacteremia (*p* = 0.06, *r* = 0.40). CRP also correlated positively with CIT on day 1 (*p* = 0.04, *r* = 0.44) and day 14 (*p* = 0.052, *r* = 0.43), but negatively on day 7 (*p* = 0.057, *r*= −0.41), indicating a complex, time-dependent relationship (Fig. S5).

### Impact of combined injury on gut microbiota

A mean of 209,780 forward reads per sample were generated from 16S amplicon sequencing. Alpha diversity metrics, Shannon and Faith PD, remained relatively stable across time in each group with no significant differences (Fig. S6A). Beta diversity metrics, Bray-Curtis (BC) and Generalized UniFrac (GU), showed no significant shifts at baseline amongst groups. On day 2, radiation led to significantly different phylogenetic clustering than burn (*p* = 0.024) and CI (*p* = 0.046) (Fig. S6B-C). Alternatively, non-phylogenetic clustering was distinct on day 3 with burn having significant alterations compared to radiation (*p* = 0.046) and CI (*p* = 0.011).

**Fig. 4:**
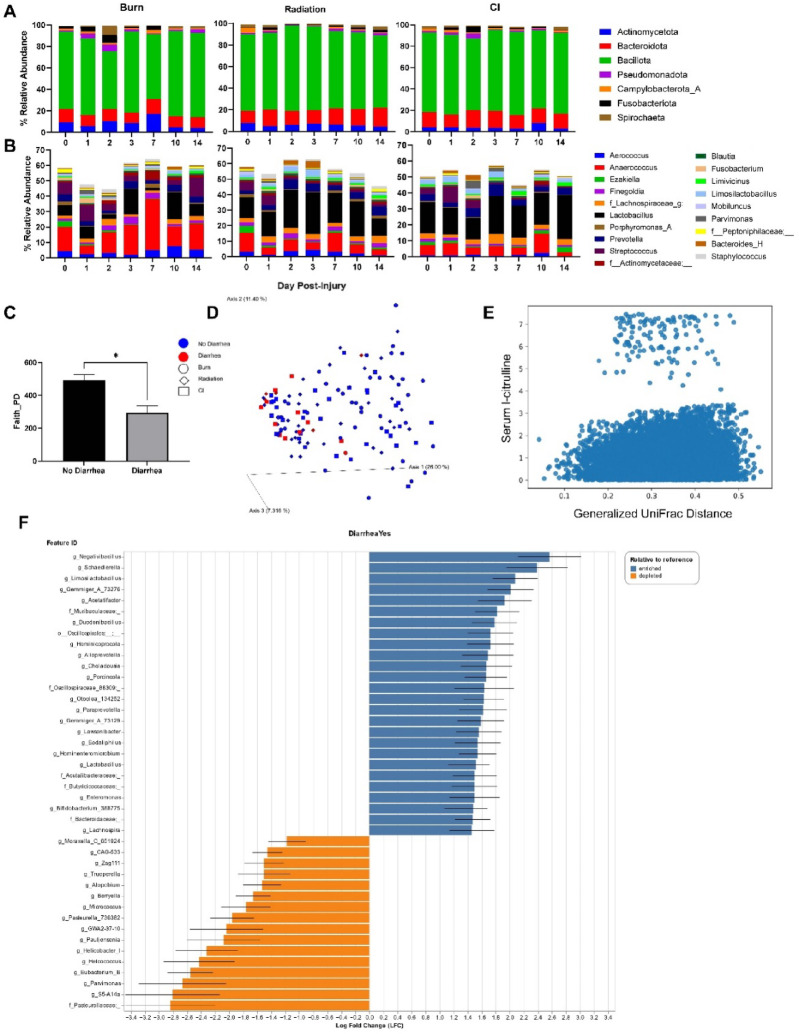
The impact of combined burn and radiation on the gut microbiota. (**A-B**) Longitudinal taxonomic classifications of phyla and genus levels, respectively, for each injury group. Barplots represent mean relative abundance. Phyla shown have mean relative abundance over 1%. The top 20 genera are represented. (**C-E**) Correlation of diarrhea prevalence and circulating biomarkers of intestinal dysfunction with bacterial diversity. (**C**) Barplot representing difference in Faith’s phylogenetic alpha diversity in the presence or absence of diarrhea is shown. *P < 0.05 (One-way ANOVA, Tukey’s post-testing). (**D**) Generalized UniFrac analysis evaluating microbial community composition and diarrhea prevalence. Each axis of the PCoA plot explains the variance in the data. Each dot (burn), diamond (radiation), or square (CI) is an individual sample. Red color-coded samples represent the presence of diarrhea whereas blue is the lack thereof. (**E**) Shows correlation between Generalized UniFrac and serum l-citrulline. Correlation was calculated by Mantel test with points representing each animal and timepoint. (**F**) Significantly differential abundant genera with log fold change represented with genera either enriched (blue) or depleted (orange) in the presence of diarrhea. Burn (d0, *n* = 7; d1, *n* = 6; d2, *n* = 5; d3, *n* = 6; d7, *n* = 3; d10, *n* = 6; d14, *n* = 6), radiation (d0, *n* = 6; d1, *n* = 7; d2, *n* = 7; d3, *n* = 7; d7, *n* = 5; d10, *n* = 7; d14, *n* = 6), CI (d0, *n* = 6; d1, *n* = 7; d2, *n* = 6; d3, *n* = 7; d7, *n* = 6; d10, *n* = 8; d14, *n* = 5).

At phyla level, Pseudomonadota significantly increased in the first 48 h post-burn (d0–d2, *p* = 0.029, LFC = 1.47) and at day 2, were significantly higher in both CI (*p* = 0.0087) and burn (*p* < 0.0001) compared to radiation (Fig. 4A). Fusobacteriota increased in all groups within 48 h post-injury, with a significant rise observed on day 2 following CI (*p* = 0.049, LFC = 3.96), and also higher abundance post-burn compared to radiation (*p* = 0.015). At the genus level, the burn group showed notable variability resulting in dramatic relative abundance shifts over time, while the radiation and CI groups exhibited more stable longitudinal composition (Fig. 4B). Using ANCOM-BC, we identified 69 differentially abundant genera across groups longitudinally, which included 8 genera post-burn group, 23 post-irradiation, and 40 post-CI, with the most genera observed in the CI group at day 7 and 14 (Table S2).

We next evaluated the gut microbiota in the context of intestinal dysfunction. While no significant associations were found with Shannon diversity, Faith PD was significantly lower when diarrhea was present (*p* = 0.017) (Fig. 4C), and also negatively correlated with iFABP levels (*p* = 0.016, *r*= −0.213). Both GU and BC diversity metrics revealed significant community differences associated with diarrhea (*p* = 0.001, Fig. 4D). Mantel testing demonstrated a significant correlation between CIT and phylogenetic beta diversity (GU, *p* = 0.01, Fig. 4E). Examination of taxa associations revealed significant increases in many bacterial genera with diarrhea including *Alloprevotella* (*p* = 0.0017), *Paraprevotella* (*p* = 0.0009), *Bifidobacterium* (*p* = 0.003), and *Lactobacillus* (*p* = 0.0008). There were significant reductions in *Helicobacter* (*p* < 0.0001), *Parvimonas* (*p* = 0.0008), and *Eubacterium* (*p* < 0.0001) in those with diarrhea (Fig. 4F). Longitudinal and terminal biomarker correlations showed that CIT exhibited significant positive correlation with seven different genera, while i-FABP had a single significant correlation (Table S3).

### Liver dysfunction and differential gene expression following combined injury

**Fig. 5:**
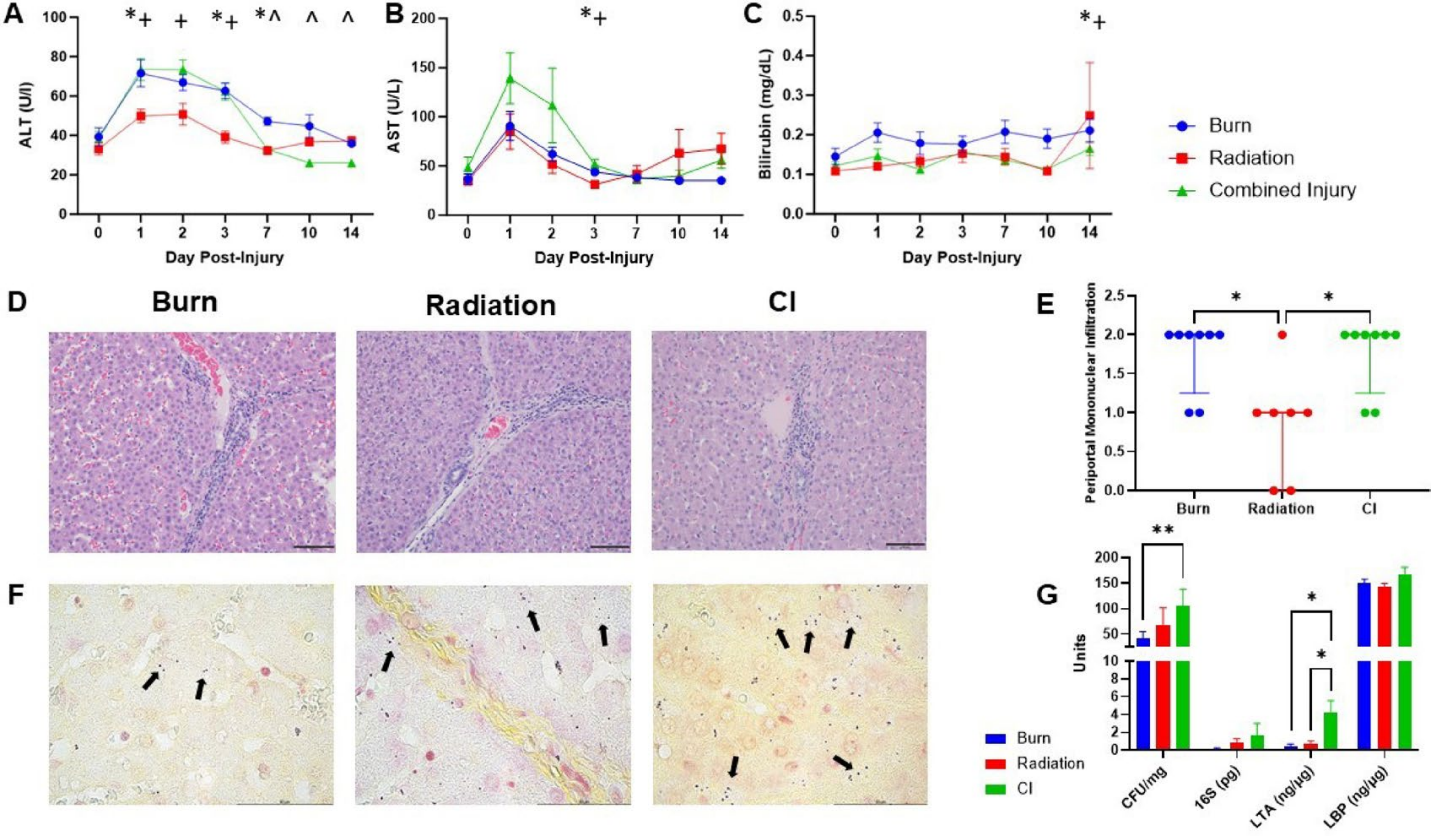
Liver chemistries, histology, and bacterial translocation. (**A**) ALT, (**B**) AST, and (**C**) Bilirubin. Two-way ANOVA with post-hoc testing revealed a * significant difference between burn and radiation, ^ significance difference between burn and CI, and + significance difference between radiation and combined injury. (**D**) Representative H & E stained liver slides from each injury pattern (scale bar 100 μm). The color of each symbol represents the injury group. (**E**) Blinded pathologist scoring of peripheral mononuclear cell and neutrophil infiltration. *P* < 0.05 (Kruskal-Wallis). (**F**) Representative images of modified Gram-stained liver sections from each injury group, purple represents Gram-positive organisms whereas Gram-negative organisms are pink. Scale bar at 50 μm. (**G**) Bargraphs showing bacterial growth and products into the liver at day 14. Countable range from tissue, 15–150 bacterial colonies. Detection of 16S rRNA concentration in liver tissue using 16S rRNA qPCR. Liver lipoteichoic acid (LTA) and lipopolysaccharide-binding protein (LBP) levels determined by ELISA on day 14 (ng/µg). Statistical significance was determined using two-way ANOVA. Two-way ANOVA revealed significant effect of injury pattern (*P* = 0.046) with post-hoc testing indicated as * *P* < 0.05, ** *P* < 0.005. Burn (*n* = 8), radiation (*n* = 7), and CI (*n* = 8) for each timepoint.

To explore hepatic dysfunction, we assessed levels of liver enzymes alanine and aspartate aminotransferases (ALT and AST) and bilirubin, which revealed a profound increase in both enzymes, with significantly higher elevations in the burn and CI groups (Fig. 5A-C). Pathologist analysis of the liver showed increased infiltration of mononuclear cells with radiation compared to burn (*p* = 0.035) and CI (*p* = 0.035) (Fig. 5D-E). The livers also showed significant levels of culturable bacteria, which was significantly higher in CI (*p* = 0.003). CI also showed a non-significant increase in 16S DNA levels (*p* = 0.44–0.75), and significantly increased lipoteichoic acid (Gram-positive cell component) (*p* = 0.013–0.026) but not lipopolysaccharide binding protein (LBP, Gram-negative marker) (Fig. 5F-G). This was corroborated with modified Gram staining of the liver, which showed that most Gram-positive bacteria were cocci. We also observed gut-liver correlations with jejunal occludin levels and liver CFU/mg (*p* = 0.018, *r*= −0.55). Furthermore, liver LBP levels correlated positively with circulating CRP (*p* = 0.016, *r* = 0.52), jejunal NLRP3 (*p* = 0.043, *r* = 0.456) and Caspase-1 (*p* = 0.035, *r* = 0.472), and liver CFU/mg to jejunal IL-1β (*p* = 0.06, *r* = 0.449) (Fig. S6).

**Fig. 6:**
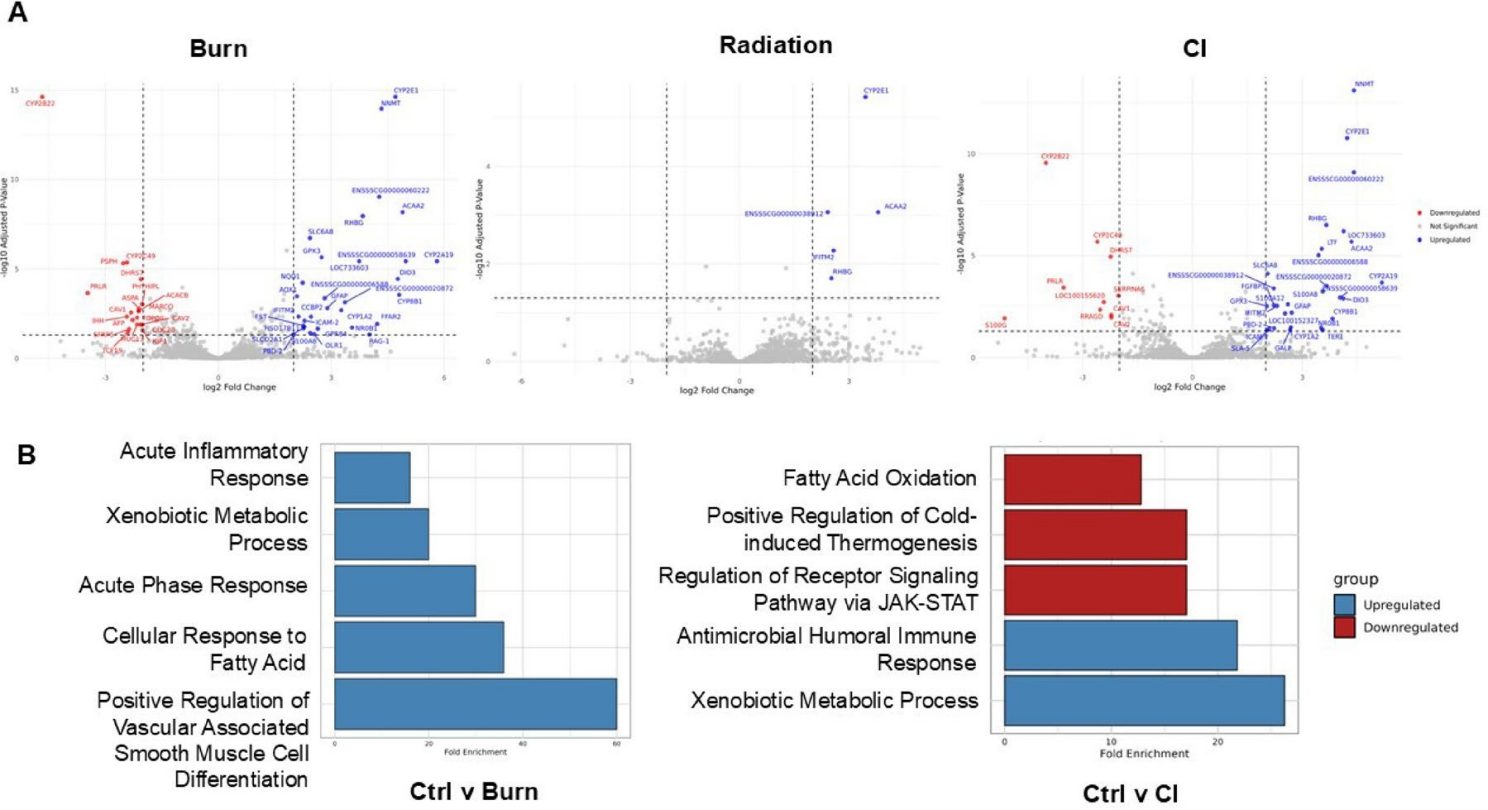
Differentially expressed genes in the liver of burn, radiation, and CI minpigs compared to uninjured controls. (**A**) Volcano plots showing the changes of liver genes compared to control in each injury group (adjusted *p* < 0.05 value, log fold change ≥ 2), blue represents downregulated genes and upregulated genes are in red; (**B**) Gene Ontology (GO) biological process analysis reveals the biological functions that are enriched in the significantly up-and down-regulated genes. Control versus both burn and combined injury. Control (*n* = 6), burn (*n* = 8), radiation (*n* = 6), and CI (*n* = 7).

Thus, liver RNA sequencing with differential expressed gene (DEGs) analysis (FDR cutoff = 0.05, log2fold change ≥ 2) revealed that burn injury led to the most significant alterations in gene expression post-injury (Fig. 6A). The highest number of changes were between burn and radiation (143 total, 116 upregulated and 27 downregulated), followed by control-burn (118 total, 59 upregulated and 59 downregulated). Comparisons involving CI showed fewer changes, with 69 total DEGs from control and only 22 compared to burn. Radiation compared to control and CI displayed the least DEGs, with 6 and 14, respectively. Gene Ontology (GO) biological process analysis identified significantly enriched pathways post-burn related to smooth muscle cell differentiation, fatty acid cellular response, and inflammation. Additionally, post-CI upregulated pathways included xenobiotic metabolic processes and antimicrobial humoral immune response while downregulated pathways were associated with regulation of receptor signaling pathway via JAK-STAT, regulation of thermogenesis, and fatty acid oxidation. Irradiation did not induce any significantly altered biological processes in the liver on day 14 (Fig. 6B), revealing that burn influenced hepatic gene expression much more than irradiation.

### Correlation of CI induced gut microbiota with liver biomarkers and DEGs

**Fig. 7:**
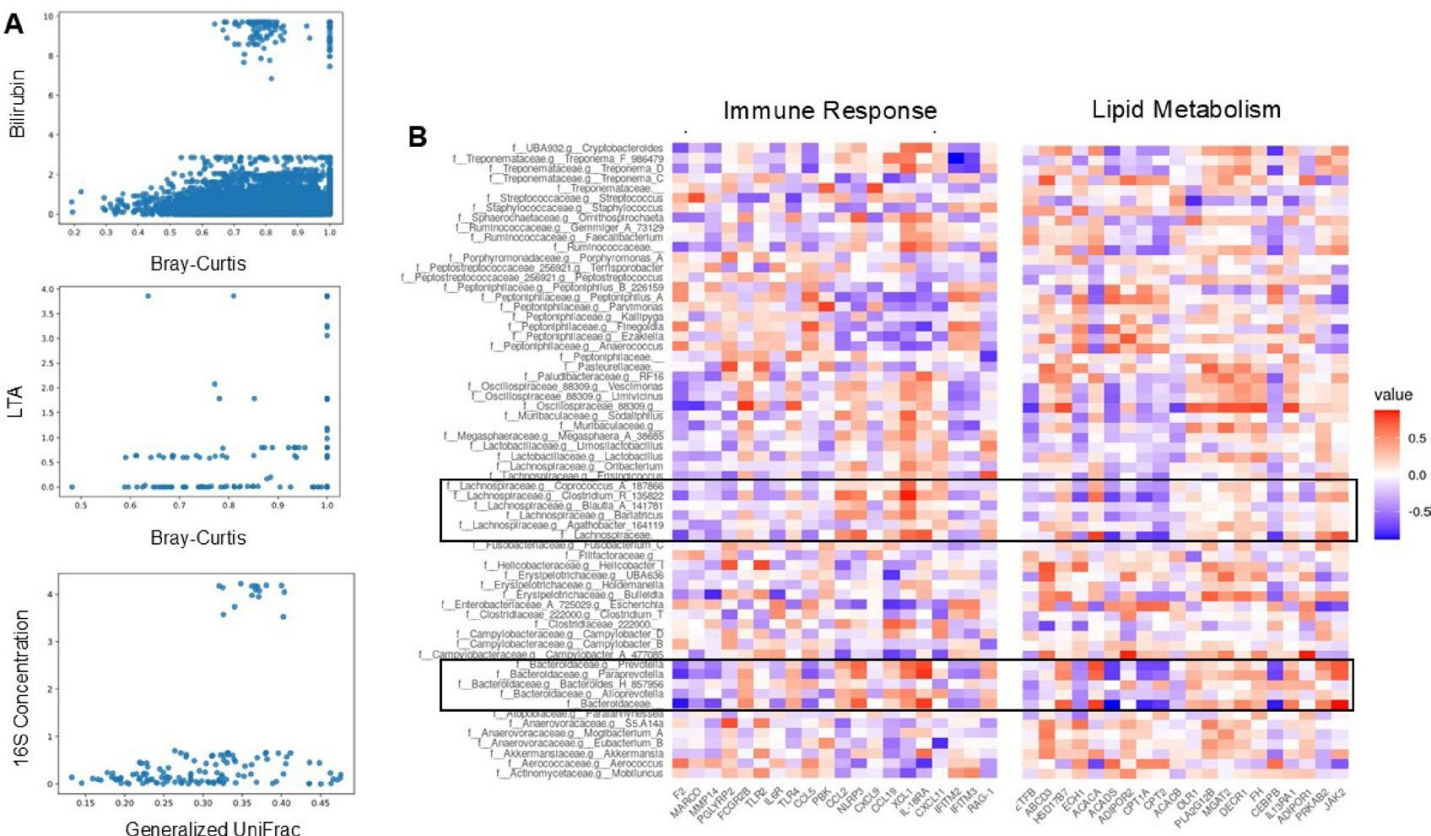
Associations between gut microbiota and liver function. (**A**) Mantel testing reveals correlation between beta diversity metrics (Bray-Curtis or Generalized UniFrac; *n* = 129) and circulating bilirubin (*n* = 161), liver lipoteichoic acid (LTA; *n* = 23, and liver 16S concentrations (*n* = 23). (**B**) Heatmaps of the top 20 rlog transformed gene levels involved in immune response and lipid metabolism compared to genera with prevalence > 0.5%. 16 S data (*n* = 129) and liver gene expression (*n* = 27).

We evaluated the gut microbiome and host liver gene expression relationship induced by injury. No significant correlations were observed between alpha diversity metrics and circulating liver enzymes (data not shown). However, a strong correlation was identified between Bray-Curtis beta diversity and microbial communities with bilirubin levels (BC, *p* = 0.011; Fig. 7A). We also noted weak correlations for ALT and AST with beta diversity (BC; ALT: *p* = 0.14, AST: *p* = 0.20). Furthermore, on day 14, beta diversity was correlated with liver 16S levels (GU, *p* = 0.043) and LTA (BC, *p* = 0.042).

We then screened the 163 DEGs with genera relative abundance greater than 0.5%, followed by analysis of significant differences of enriched pathways shown in Fig. 6B. Ultimately, correlation of the top 20 DEGs related to immune response and lipid metabolism and prevalent genera are represented (Fig. 7B). We observed significant gene-bacterial correlations between *Bacteroidaceae* and *Lachnospiraceae* family members associated with immune response. The most significant interactions with immune response genes were seen with *IL-18RA* (*n* = 6) and *F2* having three and *CCL19* two each. Interestingly, *Escherichia* (*p* = 0.039) and *Campylobacter* (*p* = 0.049) were negatively correlated with *CCL19* gene expression. Furthermore, *Bacteroidaceae* and *Lachnospiraceae* family members also exhibited significant associations with lipid metabolism-related genes: *ACACA* (*n* = 3), *ACADS* (*n* = 4), and *CEBPB* (*n* = 3) (Table S4).

## Discussion

A nuclear/radiological mass casualty event would cause various injury patterns including a high incidence of combined thermal burns and radiation exposure. Given the systemic effects of burn and irradiation, damage to individual organ systems will contribute to profound critical illness and MOD. To our knowledge, we present the first large animal CI model with MOD that focuses on GI-ARS and how burn and radiation-induced injury impact intestinal permeability, bacterial translocation, and gut microbiota changes. Additionally, this study is the first to investigate the gut microbiome-liver axis following CI. The key findings demonstrate that CI resulted in increased jejunal NLRP3 inflammasome activation, decreased barrier function, gut microbiota alterations, and perturbed liver gene expression. Additionally, the gut-liver axis was highlighted by correlations between gut microbiota abundance and liver biological process pathways. These findings provide novel insights into the systemic consequences of CI and may offer new therapeutic targets for mitigating MOD in burns, radiation, and CI.

Previous research shows that combined burn and radiation exacerbates mortality marked by acute inflammation, intestinal apoptosis, disrupted gut epithelium, and bacterial translocation^19–21^. While our results align with these CI models, a notable difference is the absence of increased mortality due to the partial body exposure and lower dose of irradiation we used^19,20^. Timing of the injury pattern is also an important consideration as Ledney et al. (1981) found that wounding prior to radiation exposure improves survival compared to irradiation before wounding^22^. Additionally, our terminal timepoint of 14 days may explain why CI did not increase jejunal villi blunting or apoptosis which has been shown previously. Of note, Carter et al. (2016) reported reduced intestinal recovery within 72 h following CI; thus, time following injury is crucial^21^. Interestingly, although radiation did not induce appreciable neutropenia, burn-induced increases in neutrophils were delayed by the addition of radiation in CI until day 7, when they began to increase towards levels seen in burn alone. This differs from Palmer et al. (2011) which documented acute neutrophil elevations and persistently low lymphocyte levels^19^. These differences highlight the influence of injury order and timing of tissue collection on outcomes.

Burn and radiation both induce MOD through distinct mechanisms, affecting organs to varying degrees. Burn-induced MOD depends on size, depth, and timing, as the acute hypometabolic ebb phase gives way to the hypermetabolic flow phase that includes hepatic lipid accumulation, hepatomegaly, and hyperglycemia, as well as ER stress and mitochondrial damage contributing to hepatocyte apoptosis^3,23^. Burns also frequently cause acute kidney injury due to early hypoperfusion and later inflammation-induced renal damage^3,23^.

In contrast, radiation-induced MOD depends on dose, exposure area, and radiation quality, primarily affecting organs with rapidly dividing cells (e.g., hematopoietic, GI)^4^. Vascular endothelium damage leads to barrier loss, tissue edema, and hypoxia, while immune cell depletion promotes chronic immunosuppression^4,24^. In our model of partial body exposure, sufficient bone marrow sparing prevented the contribution of hematopoiesis deficits on immune suppression, as evidenced by the lack of leukopenia in the radiation alone group. While DNA damage and associated oxidative stress is the main mechanism studied in ARS^4,24^, this supports a role for vascular damage and GI permeability increases in the immune response in our model. In terms of MOD, ARS-related liver and kidney injury remain relatively unexplored, typically assessed through clinical chemistries^25,26^ by day 14 in our model burn injury caused more liver inflammation compared to radiation, whereas radiation had a greater impact on kidney injury. These findings suggest that, despite some functional recovery, persistent inflammation and tissue damage may contribute to long-term MOD, highlighting distinct organ-specific effects of each insult. Alterations in the gut microbiota (i.e., dysbiosis), characterized by an imbalance in microbial diversity or commensal flora, often correlates with increased inflammation, impaired intestinal barrier function, and susceptibility to infections^27,28^. While dysbiosis is often defined as reduced alpha diversity, results remain inconsistent across burn and radiation studies^10,11,29,30^. Generally, alpha diversity tends to decrease following burn injury, whereas many (but not all) irradiation studies frequently report no changes, with many large animal models post-irradiation reporting stable alpha diversity^10,11,29–31^. We observed no significant alpha diversity differences from baseline or amongst injury groups, suggesting that this metric is less informative for characterizing dysbiosis in the context of burns, radiation, or CI. Perhaps these discordant results are influenced by experimental factors like primer selection, sequencing depth, and rarefaction^32^. Additionally, host species differences may impact response to injury. In contrast, beta diversity often shows significant shifts post-injury, especially when examining the presence of diarrhea. Interestingly, we did elucidate a relationship between phylogenetic beta diversity and l-citrulline, which has been touted as a promising GI function biomarker for assessing radiation-induced intestinal epithelial damage^33^. Altogether, beta diversity appears more indicative of GI dysfunction in traumatic injury than alpha diversity in our model.

While diversity metrics serve as valuable indicators of microbial community dynamics, taxonomic alterations provide more actionable insights. Following injury, well-documented microbial trends include an increase in pathogenic or opportunistic taxa alongside a decline in beneficial commensal organisms. This is the first report on gut microbiota dynamics following CI, which revealed unique microbial trends. Like burn and irradiation, CI resulted in increases in opportunistic pathogens, *Bacteroides* and *Fusobacterium*. However, *Lactobacillus* exhibited a biphasic response, initially decreasing within the first 48 hours post-injury before subsequently increasing. Similarly, *Streptococcus* displayed an unusual pattern, rising in abundance during the first 48 hours before declining. Other notable trends included a gradual decrease in *Finegoldia* over time and relative stability of *Prevotella*. These findings underscore the interplay between specific taxa and injury type. For example, the increase in *Enterobacteriaceae*, *Bacteroides*, and *Fusobacterium* may reflect inflammatory or dysbiotic conditions favoring opportunistic pathogens. The enrichment of genera like *Lactobacillus* and *Bifidobacterium*, typically considered beneficial, is somewhat unexpected as previous rodent models following acute injury have reported depletion of these organisms. Our findings may indicate a compensatory response to gut injury or as a protective mechanism to restore mucosal integrity or modulate inflammation rather than their typical homeostatic roles. Alternatively, stress-induced changes in gut motility, pH, or nutrient availability could create a microenvironment that favors the overgrowth of certain facultative anaerobes, including *Lactobacillus*.

In terms of the gut microbiome, alterations following burn injuries have been more studied than irradiation and have shown increased prevalence of *Enterobacteriaceae*, *Bacteroides*, and *Streptococcus*, coupled with decreases in beneficial flora such as *Ruminococcus*, *Lactobacillus*, and *Bifidobacterium*^10^. Largely, our findings post-burn agree showing increases in opportunistic pathogens such as *Enterobacteriaceae*,* Finegoldia*, *Bacteroides*, *Fusobacterium*, and acute elevations in *Parvimonas* accompanied by decreases in beneficial flora like *Peptoniphilaceae* and transient reductions in *Lactobacillus*. However, we observed increased *Prevotella*, *Lachnospiraceae* and *Streptococcus* which is at odds with earlier studies^6,10^. Gut microbiota alterations post-irradiation have been more variable, with taxonomic findings in large animal models often discordant from those in rodents^11,29–31,34^. Interestingly, irradiation has also been linked to increases in *Prevotella*, *Lachnospiraceae*, *Enterococcaceae*, and *Akkermansia*^31,35^ which also gives specific organisms for diagnostic and therapeutic targets. Moreover, our current report aligns with previous findings, showing increases in *Bacteroides*, *Fusobacterium*,* Lactobacillus*, and *Prevotella* with low abundances of *Streptococcus*^11^.

To this end, therapeutic interventions such as probiotics and fecal microbiota transplantation (FMT) have emerged as potential strategies to restore microbial balance and mitigate infectious complications after injury. While *Lactobacillus* and *Bifidobacterium* are the most commonly used, other taxa such as *Faecalibacterium*,* Akkermansia*,* Eubacterium*, and *Streptococcus* are also being explored^9,36,37^. Despite limited literature on microbial based therapeutics following burn injury, data suggest their potential for improving recovery. *Clostridium butyricum* supplementation in burned mice improved intestinal barrier integrity, while probiotic treatment in burn patients significantly increased serum IgA levels, enhancing mucosal immunity^38,39^. Similarly, FMT reduced inflammation and restored gut permeability in burned mice^40,41^.

Clinical studies in cancer patients undergoing radiotherapy also report reduced diarrhea and GI symptoms with probiotics. Notably, a synthetic *Limosilactobacillus reuteri* strain engineered to release interferon-beta preserved stem cells and barrier function during irradiation. In our study, beneficial taxa like *Eubacterium*, *Clostridium*, and *Filifactor* correlated with protective markers (e.g., l-citrulline), while *Parvimonas* correlated with injury markers (iFABP), underscoring the need for precision approaches in microbial therapeutics post-injury.

Evidence for probiotic and FMT efficacy in radiation-induced GI damage is more robust^42,43^. In rodent models, supplementation with *Lactobacillus rhamnosus* GG, *Lactobacillus indica* PCC 8005, and multi-strain formulations containing *Lactobacillus*, *Streptococcus*, and *Bifidobacterium* preserved intestinal morphology and reduced inflammation^42,43^. However, we found unexpected increases in *Bifidobacterium* and *Lactobacillus* suggesting these commercially available probiotics may not be useful in the context of radiation injury^11^. Moreover, FMT improved microbial diversity, epithelial integrity, and survival in irradiated animals^35,42,43^. In cancer patients undergoing pelvic or abdominal radiotherapy, probiotic supplementation reduced the severity and frequency of diarrhea, with a combination of strains proving more effective than single organisms^42,43^. Recently, the administration of *Limosilactobacillus reuteri* engineered to release interferon-beta during whole-abdomen irradiation enhanced intestinal barrier integrity and significantly preserved intestinal stem cell populations, leading to improved survival^44^. Although we did not evaluate specific microbial therapeutics, we observed that beneficial and supportive taxa such as *Eubacterium*, *Clostridium*, *Peptoniphilus*, and *Filifactor* were positively correlated with l-citrulline levels. In contrast, *Parvimonas*, an opportunistic pathogen, was positively correlated with circulating iFABP concentration. This highlights the complexity of the gut microbiota post-injury, where both beneficial and pathogenic organisms can shift depending on the immune response and microbial interventions. These findings underscore the need for further research to optimize strain selection with whole genome sequencing, timing of intervention, and individualized approaches for microbial-based therapies in burn and radiation injury.

Burn and radiation injuries each induce profound systemic responses, significantly affecting liver function. In the acute phase post-burn (24 hours), the liver upregulates acute-phase (FGA/B/G, CFH, C8A) and inflammatory (Lcn2, Hsp90aa1/b1, Cxcl1, S100a8/a9) genes while downregulating steroid hormone biosynthesis (CYP3A25, AKR1D1, UGT2A3/B1, HSD17B6, CYP2C, CYP1A2, CYP7A1) and xenobiotic metabolism (SULT2A1/3/5/8, GSTA2/3/4, UGT2A3/B1, CYP1A2, CYP2F2, GSTM3)^45^. Peak metabolic disruptions have been observed within 24 hours (AldoA, Eno1, Cps1, Slc38a4, Mccc2), with partial recovery by day 7, and replenishment of triglyceride reserves by day 4^46^. Inflammatory genes also peaked at 24 hours but declined after, while acute-phase genes (e.g., fibrinogen, ceruloplasmin) showed sustained upregulation until day 7^46^. However, in our model, sustained upregulation of acute-phase (e.g., *IL-18RA*,* IL12B*), inflammatory (*IL34*,* DUSP13*), and xenobiotic metabolism genes (*CYP24A1*,* PNMT*) persisted through day 14, suggesting a prolonged stress response. While prior studies report the highest inflammatory responses and metabolic shifts within the first week post-injury, our findings suggest a prolonged acute-phase and xenobiotic metabolic response. This may be influenced by differences in study design, including the presence of eschar, which could delay resolution of inflammation and metabolic recovery.

Radiation similarly induces a cascade of genomic changes in the liver marked by DNA damage response activation (Cdkn1a, Phlda3, Eda2r), detoxification (Cyp2b22, Gsta2, Mgst3), lipid metabolism (Acaca, Acacb, Scd1, Lpin1), and immune modulation (Il1r1, Cd40, Irf5)^47,48^. Liver DEGs observed in a high-dose total body irradiation (TBI) mouse model at 48 hours showed significant overlap with that of a low-dose TBI Gottingen minipig model over 45 days. However, we did not observe any pathways significantly altered on day 14 post-irradiation with upregulation of CYP2E1, ACAA2, IFITM2/3, and RHBG.

In contrast, CI animals exhibited downregulation of fatty acid oxidation (CYP2C49, CYP2B22, DHRS7, CAV1), positive regulation of cold-induced thermogenesis (CAV2, S100G), regulation of receptor signaling pathway via JAK-STAT as well as upregulation of antimicrobial humoral immune response mediated by antimicrobial peptides (NNMT, CRP, S100A9, S100A8, IFITM2, IFITM3, S100A12, PBD-2, LTF) and xenobiotic metabolic processes (CYP2E1, CYP1A2, CYP2A19, ACAA2, CYP8B1, GPX3, RHBG, PDZK1IP1, TERT). These alterations suggest ongoing microbial translocation, potentially driven by taxa like *Fusobacterium* and *Bacteroides*, and impaired mitochondrial function, leading to compensatory activation of thermogenesis pathways and sustained inflammation^49–51^.

We were also able to make specific correlates to gut flora (Fig. 7B) that include both IL18RA with increased *Lachnospiraceae* (with potential downstream effects on liver CFU/LTA and C-reactive protein) and NLRP3 with increased *Bacteroidaceae* (with potential downstream effects on gut permeability and ensuing liver AST and bilirubin). Collectively, these findings highlight the importance of the gut-liver axis and the effects of CI on mitochondrial function, lipid metabolism, and immune regulation.

The interplay between the gut microbiota and liver function has garnered increasing attention. Emerging evidence suggests that gut-liver crosstalk plays a critical role in modulating inflammatory responses, metabolic pathways, and immune signaling following acute injuries^6^. We see clear increases in liver culture following CI, indicating a link between gut alterations and the liver. This has been shown in burns alone as well, with a previous study finding liver enzymes correlating to bacterial beta diversity^6^. We also observed this with bilirubin correlations with bacterial diversity. *Bacteroides*, *Prevotella*, and *Enterobacteriaceae* were associated with chronic liver conditions^52^. Additionally, Muroaka et al. (2020) show these organisms with *Veillonella*, *Fusobacterium*, and *Pasteurella* enriched post-burn in liver dysfunction^6^. We observed significant gene-bacterial correlations between *Bacteroidaceae* and *Lachnospiraceae* family members associated with immune response and lipid metabolism. Together, these findings emphasize the interconnected nature of gut and liver health, suggesting that microbial changes can exacerbate or modulate liver injury and recovery processes in the context of burn and radiation exposure.

We acknowledge several limitations in our model, including the exclusive use of male swine and a limited sample size. Moreover, the selection of day 14 for tissue collection may not have been ideal for looking at GI dysfunction, as clinical symptoms were more profound in the first week. Additionally, a larger burn or higher radiation dose would have addressed questions about mortality after CI. Finally, rectal swabs were used to assess the gut microbiota which may not fully capture luminal or mucosal communities.

Our study supports the hypothesis that CI exacerbates GI dysfunction and hepatic inflammation through disruption of the gut-liver axis and highlights the complex interplay between burn and radiation. We provide valuable insights into the longitudinal response to combined injury, linking gut microbiota alterations to differential liver gene expression. Moreover, the correlation of the gut microbiome with diarrhea, GI biomarkers, and liver functional pathways has identified specific organisms that may serve as potential diagnostic and therapeutic targets. Further investigation is required to uncover the mechanisms driving injury synergism—or the lack thereof—following combined injury, as well as to develop potential therapeutic strategies.

## Supplementary Information

Below is the link to the electronic supplementary material.

Supplementary Material 1

Supplementary Material 2

Supplementary Material 3

Supplementary Material 4

Supplementary Material 5

Supplementary Material 6

Supplementary Material 7

Supplementary Material 8

Supplementary Material 9

Supplementary Material 10

Supplementary Material 11

Supplementary Material 12

Supplementary Material 13

Supplementary Material 14

Supplementary Material 15

## Data Availability

The datasets analyzed for the current study available from the corresponding author ([david.burmeister@usuhs.edu](mailto: david.burmeister@usuhs.edu)) on reasonable request or are included in this published article and its supplementary information files. Fastq files can be found under the NCBI BioProject PRJNA1276480, https://www.ncbi.nlm.nih.gov/bioproject/?term=1276480.
