# Supplementary figures and images for: A multi-omics approach exploring the gut-liver axis following combined radiation exposure and burn injury in a Sinclair minipig model
Source: Sci Rep. 2025 Nov 20;15:41111. doi: 10.1038/s41598-025-24946-0 (PMC12635091; doi:10.1038/s41598-025-24946-0)

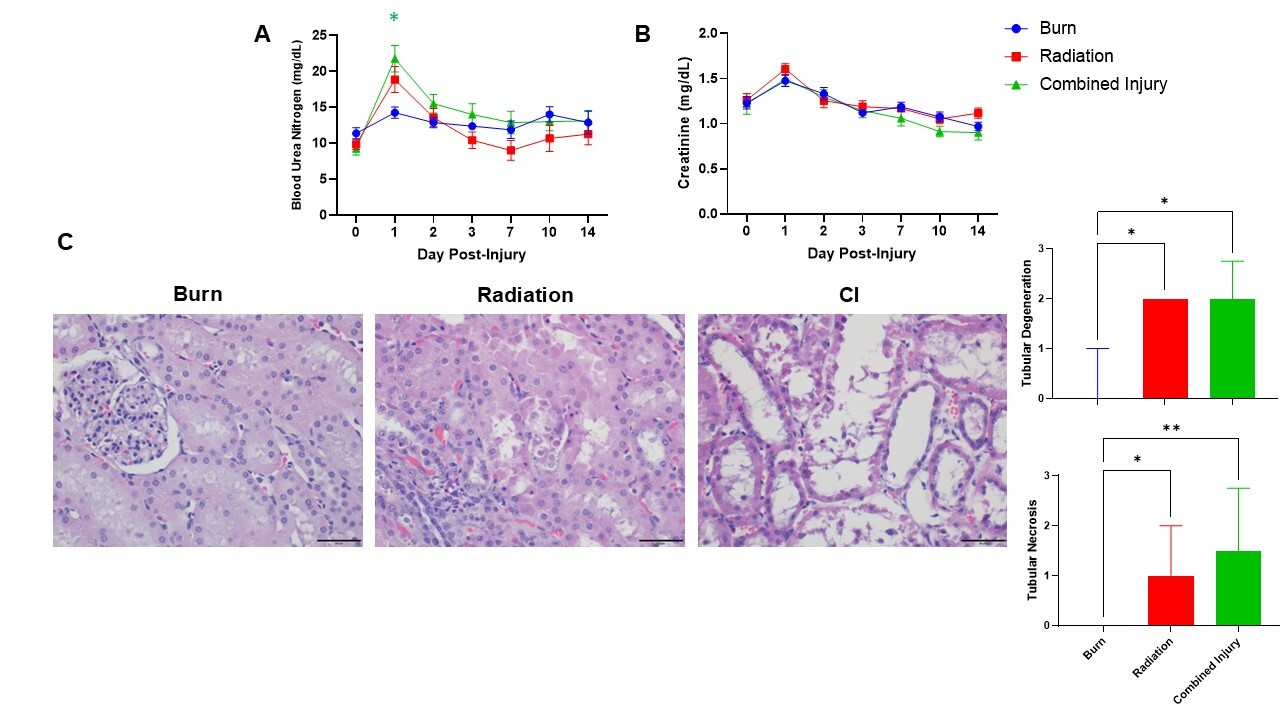

Supplement: Supplementary file 5 — Supplementary Material 5 [file 41598_2025_24946_MOESM5_ESM.jpg]

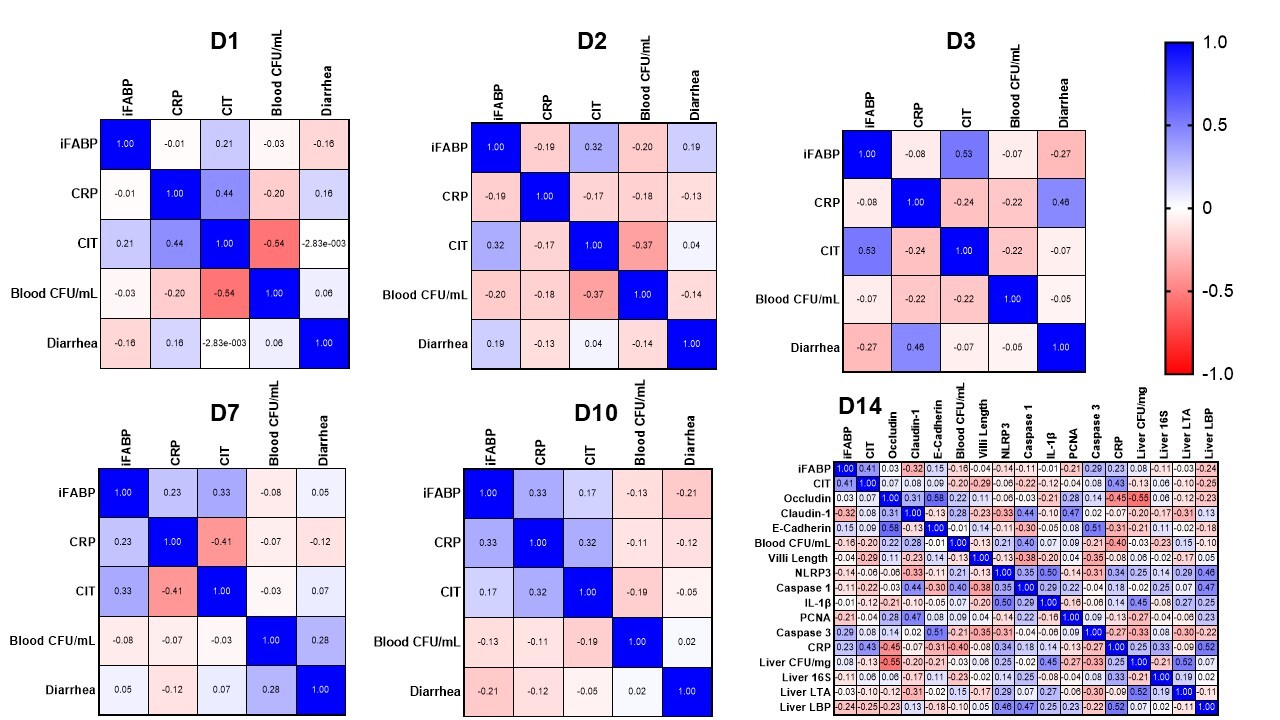

Supplement: Supplementary file 6 — Supplementary Material 6 [file 41598_2025_24946_MOESM6_ESM.jpg]

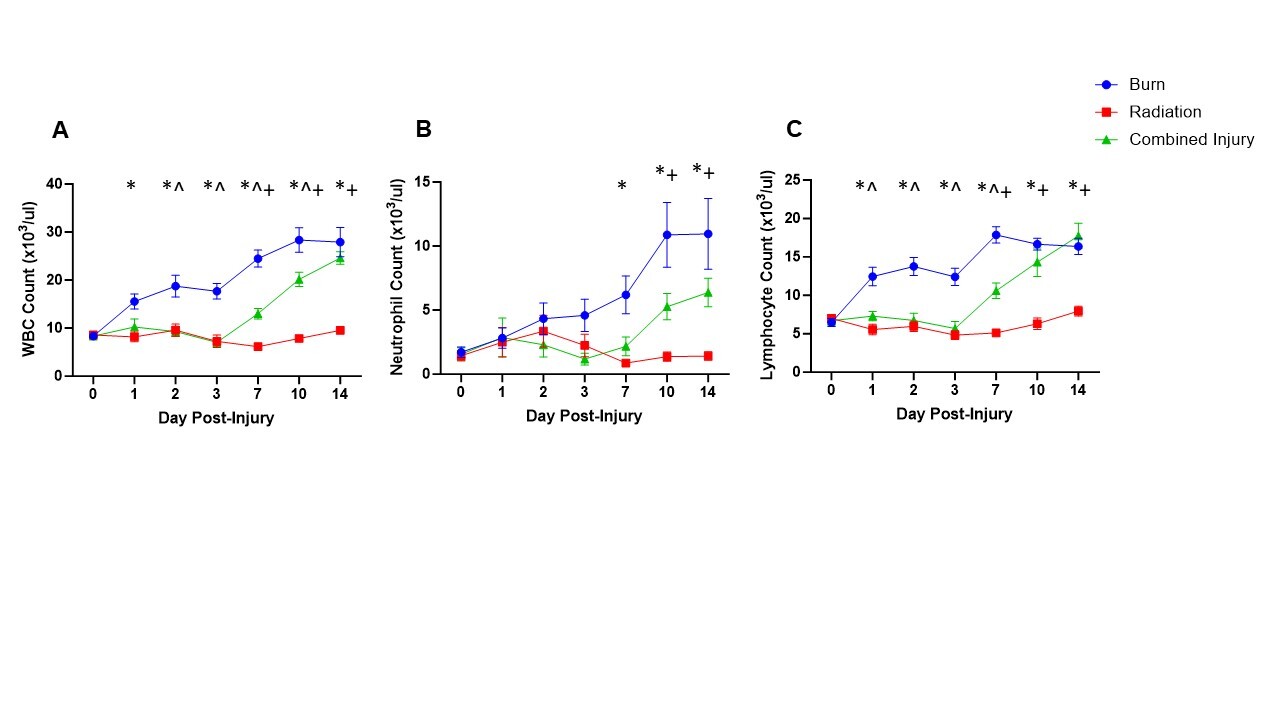

Supplement: Supplementary file 7 — Supplementary Material 7 [file 41598_2025_24946_MOESM7_ESM.jpg]

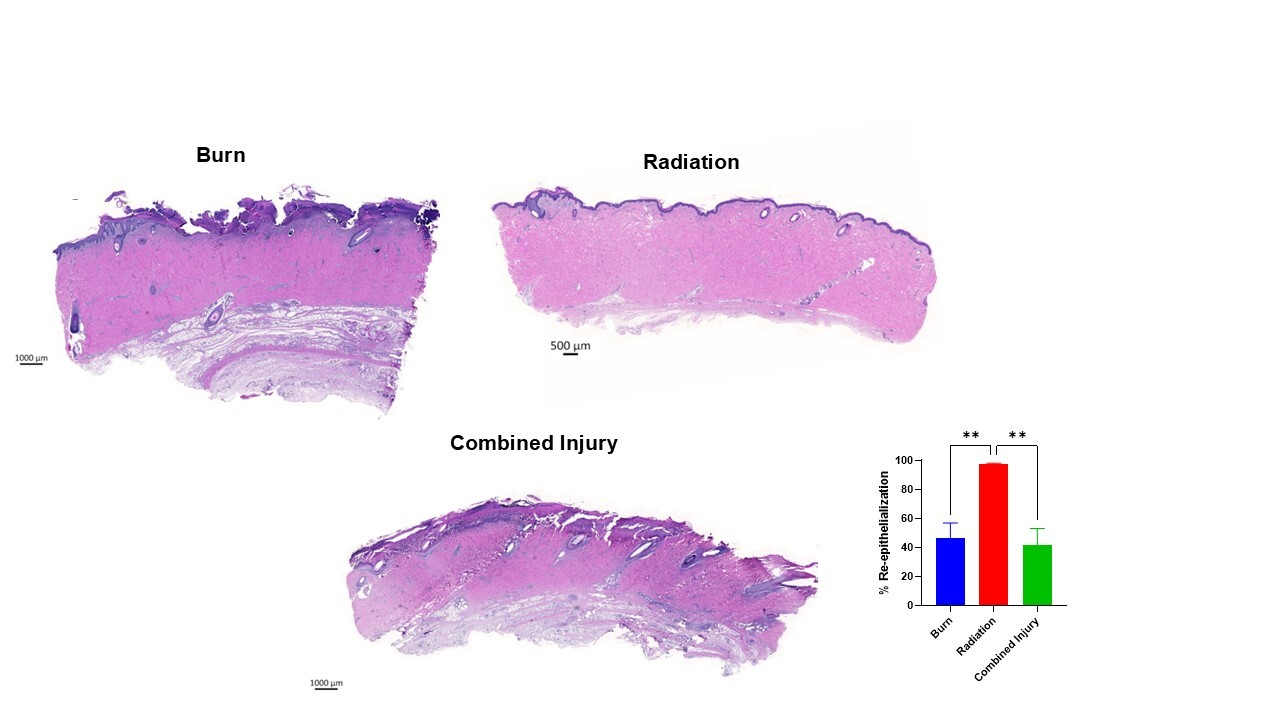

Supplement: Supplementary file 8 — Supplementary Material 8 [file 41598_2025_24946_MOESM8_ESM.jpg]

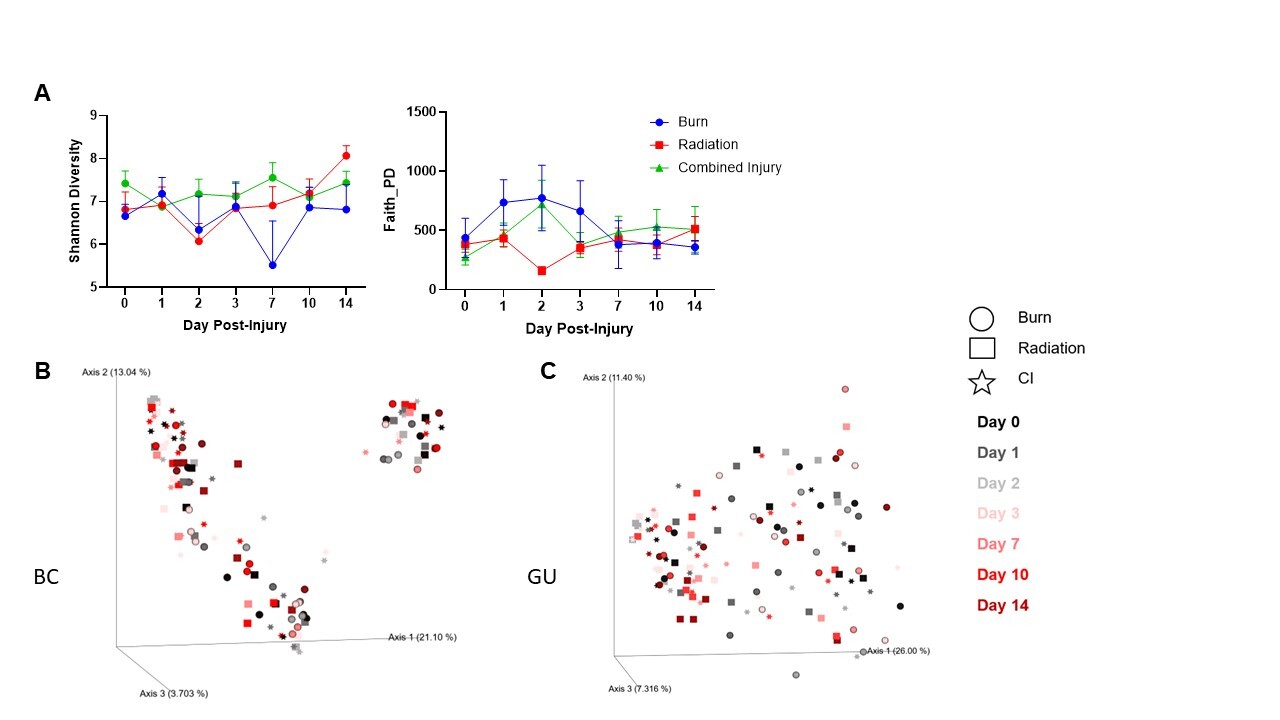

Supplement: Supplementary file 9 — Supplementary Material 9 [file 41598_2025_24946_MOESM9_ESM.jpg]

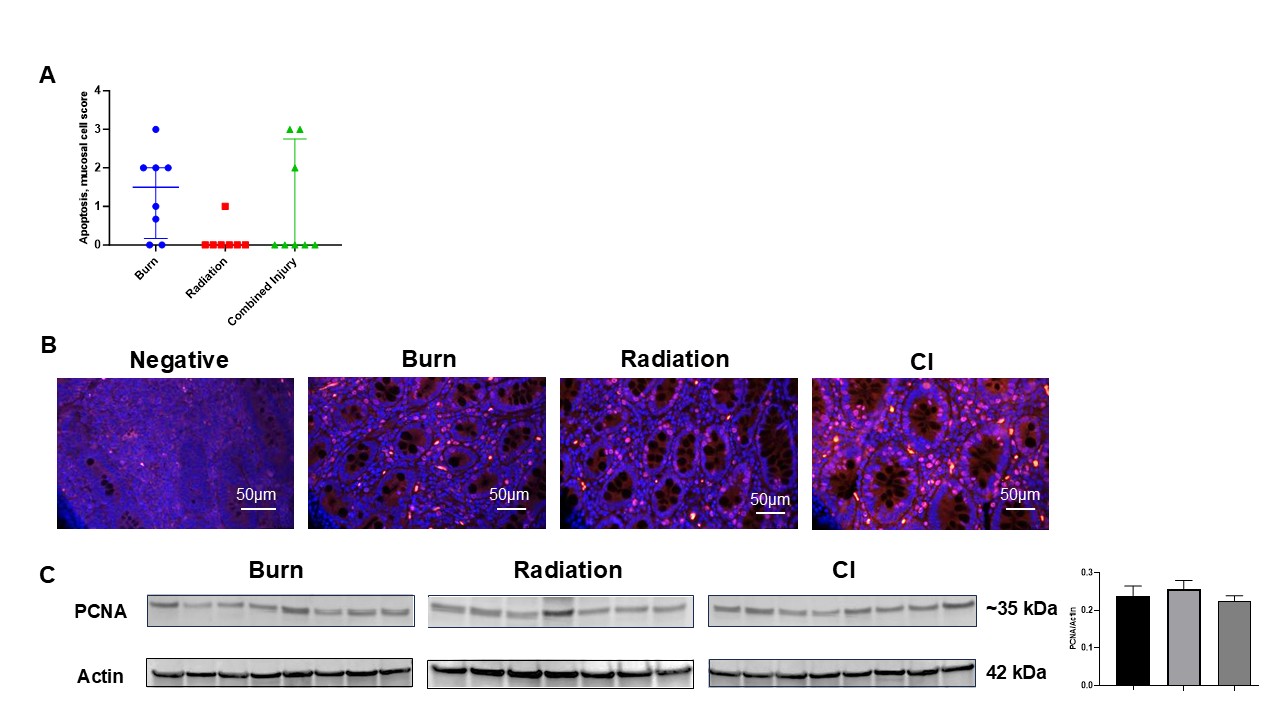

Supplement: Supplementary file 10 — Supplementary Material 10 [file 41598_2025_24946_MOESM10_ESM.jpg]

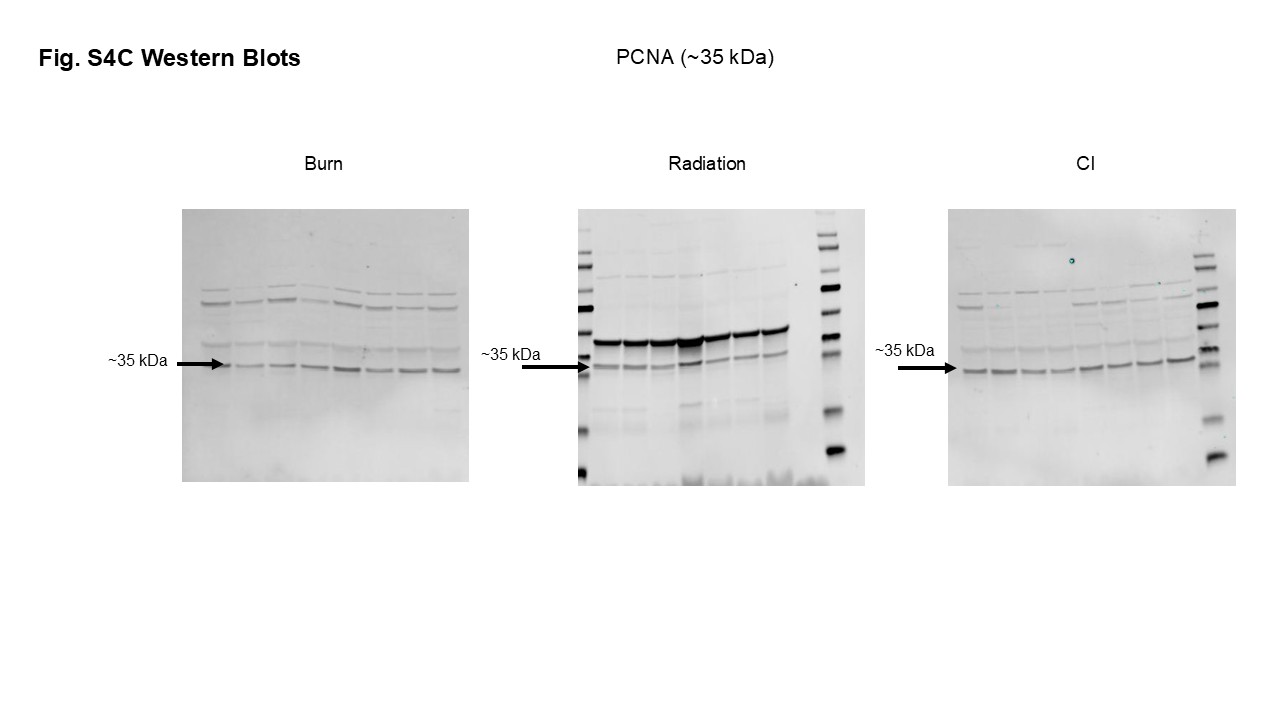

Supplement: Supplementary file 12 — Supplementary Material 12 [file 41598_2025_24946_MOESM12_ESM.jpg]

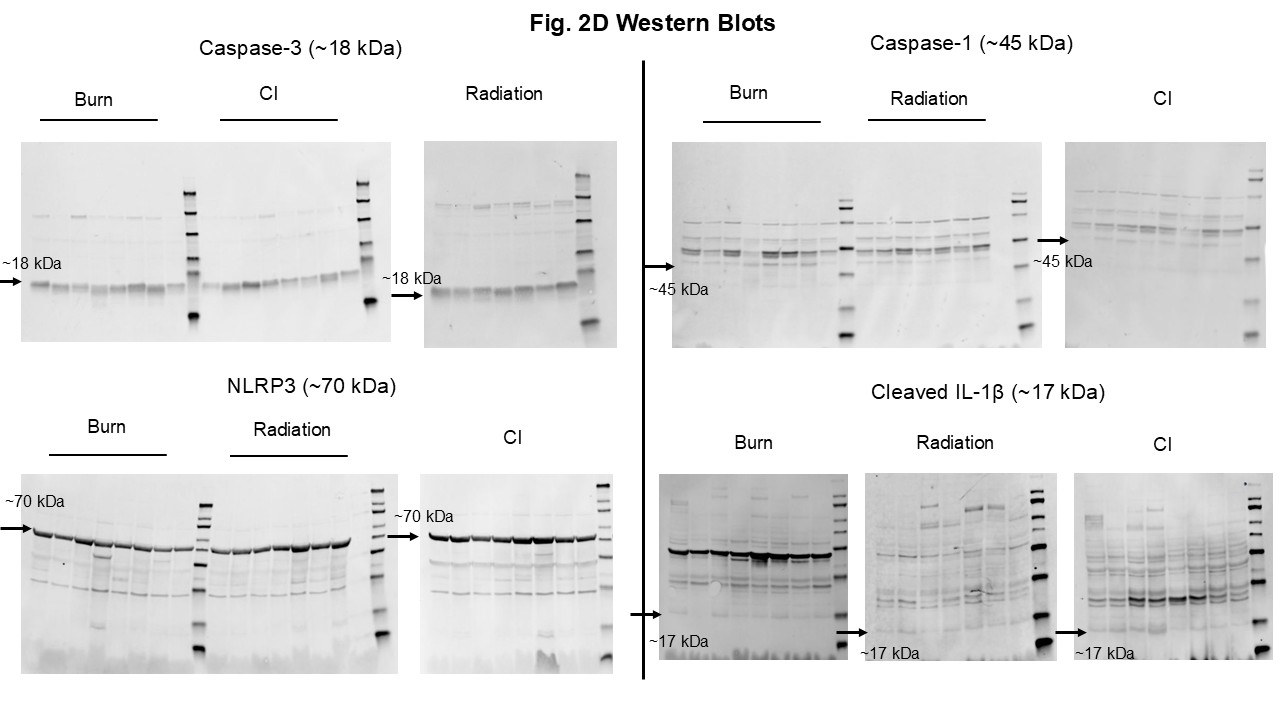

Supplement: Supplementary file 13 — Supplementary Material 13 [file 41598_2025_24946_MOESM13_ESM.jpg]

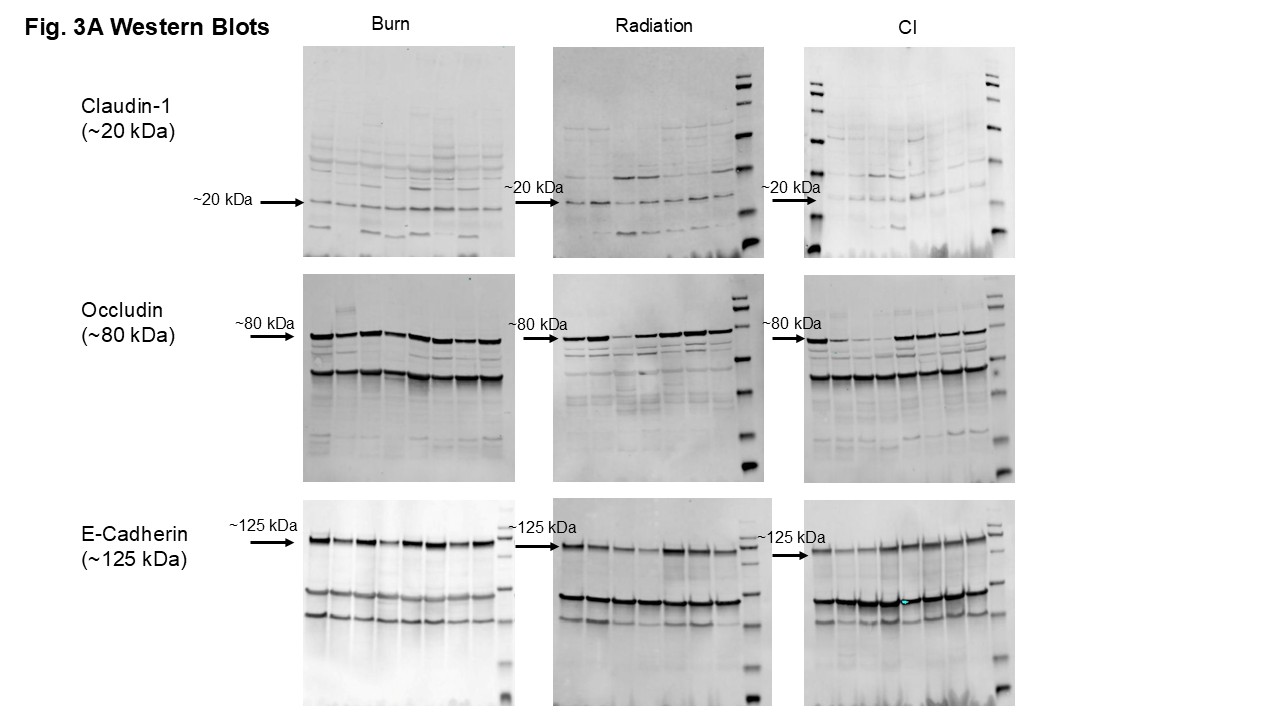

Supplement: Supplementary file 14 — Supplementary Material 14 [file 41598_2025_24946_MOESM14_ESM.jpg]

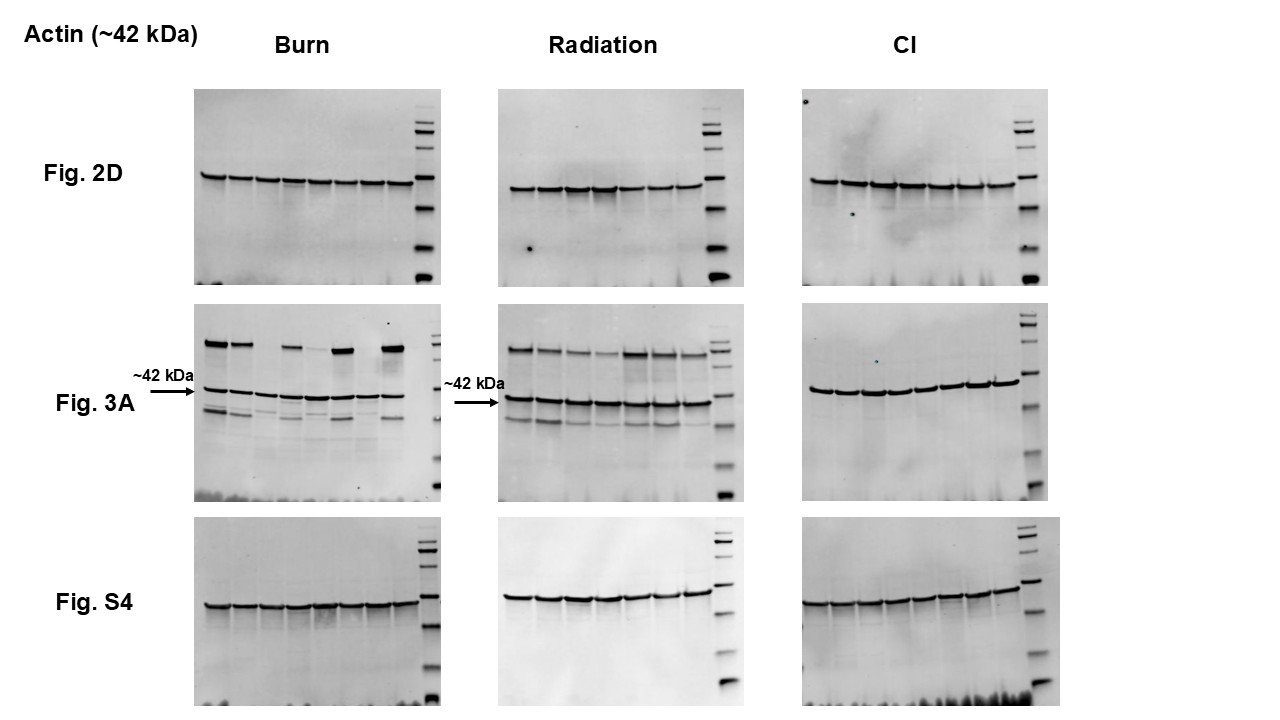

Supplement: Supplementary file 15 — Supplementary Material 15 [file 41598_2025_24946_MOESM15_ESM.jpg]
