# Supplementary material for: A multi-omics approach exploring the gut-liver axis following combined radiation exposure and burn injury in a Sinclair minipig model
Source: Sci Rep. 2025 Nov 20;15:41111. doi: 10.1038/s41598-025-24946-0 (PMC12635091; doi:10.1038/s41598-025-24946-0)
Supplement: Supplementary file 11 — Supplementary Material 11 [file 41598_2025_24946_MOESM11_ESM.docx]

**Supplemental Table and Figure Legends**

**Supplemental Table 1.** The table presents the primary antibodies used for Western blotting including their target proteins, supplier information, catalog numbers, and dilution factors. Antibodies targeting apoptosis-related proteins (Caspase-1, Caspase-3), tight and adherens junction proteins (Claudin-1, E-cadherin, Occludin), inflammatory markers (IL-1β, NLRP3), and a proliferation marker (PCNA) were obtained from various commercial sources.

**Supplemental Table 2.** Presents the differentially abundant bacterial genera identified in each injury group over time with baseline (day 0) serving as the reference for comparison. The analysis shows the injury group, bacterial genera, log fold change, and adjusted p-values calculated using Analysis of Compositions of Microbiomes with Bias Correction (ANCOM-BC). Burn (n=39), radiation (n=45), and CI (n=45).

**Supplemental Table 3.** Significant correlations between circulating biomarkers and bacterial genera. Abbreviations: CIT = l-citrulline and iFABP = intestinal fatty-acid binding protein. Analyzed using Pearson correlation. Displays the correlation coefficient (r value) and adjusted p-value. 16S data (n=129) and longitudinal biomarkers (n=161).

**Supplemental Table 4.** Correlations between bacterial genera and liver DEGs on day 14 post-injury. Table shows correlation coefficients (r-value) were calculated by Spearman correlation and indicated adjusted p values. 16S data (n=129) and liver gene expression (n=27).

**Supplemental Figure 1.** Representative H&E images from skin sections from each injury group on day 14, scale 500-1000μm. Barplot shows percent re-epithelization of skin in each group. Statistical significance was determined using One-way ANOVA, ** *P* < 0.005. Burn (n=8), radiation (n=7), and CI (n=8).

**Supplemental Figure 2.** Circulating complete blood counts from routine blood draws performed on days 0, 1, 2, 3, 7, 10, and 14. (A-C) White blood cell counts further stratified into neutrophil and lymphocyte counts. * Significant difference between burn and radiation, ^ significance difference between burn and CI, and + significance difference between radiation and combined injury. Significance between groups, *P* < 0.05 (Two-way ANOVA, Tukey’s post-testing). Burn (n=8), radiation (n=7), and CI (n=8) for each timepoint.

**Supplemental Figure 3.** Evaluation of kidney injury following injury. (A) Blood urea nitrogen (BUN) and (B) Creatinine over time post-injury. (C) Representative hematoxylin and eosin (H&E) stained kidney tissue sections following each injury type scored by a blinded veterinary pathologist. Scale at 50μm., **P* < 0.05 and ***P* < 0.005 (Kruskal-Wallis). Burn (n=8), radiation (n=7), and CI (n=8) for each timepoint.

**Supplemental Figure 4.** Cell turnover in the jejunum following injury. (A) Blinded veterinary pathologist scoring of crypt loss and mucosal cell apoptosis. (B) Representative images of Ki67 immunohistochemistry, Ki67 (red) with DAPI (blue). Scale at 50μm. (C) Representative blots of beta-actin (β-actin) of components of proliferating cell nuclear antigen (PCNA) (One-way ANOVA, Tukey’s post-hoc testing), Burn (n=8), radiation (n=7), and CI (n=8).

**Supplemental Figure 5.** Longitudinal heatmaps depicting correlation of circulating biomarkers, intestinal markers, bacterial products translocated to the liver, and diarrhea across different time points. Pearson correlation matrices depict associations between circulating biomarkers (iFABP, CRP, CIT) and clinical parameters (blood CFU/mL, diarrhea) at days 1, 2, 3, 7, and 10 post-injury. The day 14 heatmap includes additional biomarkers related to intestinal integrity (occludin, claudin-1, E-cadherin, villi length), inflammation (NLRP3, caspase-1, IL-1β), cell turnover (PCNA, caspase-3), and bacterial translocation (liver CFU/mg, liver 16S, liver LTA, liver LBP). The strength and direction of correlations are color-coded, with blue indicating positive correlations and red indicating negative correlations. The correlation coefficients (r values) are provided within each cell. Burn (n=8), radiation (n=7), and CI (n=8) for each timepoint.

**Supplemental Figure 6.** Effect of injury pattern on longitudinal alpha and beta diversity. (A) Shannon diversity (left) and Faith’s phylogenetic diversity (right) were measured across time (days 0, 1, 2, 3, 7, 10, and 14) post-injury to assess alpha (within sample) diversity from rectal swabs. Data are presented as mean ± SEM for each injury group. Colors and shapes indicate injury type. One-way ANOVA, Tukey’s post-testing. (B) Bray-Curtis and (C) Generalized UniFrac beta diversity metrics showing microbial community level composition with each injury pattern over time. Each axis of the PCoA plot variance of the data. Shapes represent injury type and time points are color-coded. Burn (d0, n=7; d1, n=6; d2, n=5; d3, n=6; d7, n=3; d10, n=6; d14, n=6), radiation (d0, n=6; d1, n=7; d2, n=7; d3, n=7; d7, n=5; d10, n=7; d14, n=6), and CI (d0, n=6; d1, n=7; d2, n=6; d3, n=7; d7, n=6; d10, n=8; d14, n=5).
